# DNA metabarcoding of littoral hard-bottom communities: high diversity and database gaps revealed by two molecular markers

**DOI:** 10.7717/peerj.4705

**Published:** 2018-05-04

**Authors:** Owen S. Wangensteen, Creu Palacín, Magdalena Guardiola, Xavier Turon

**Affiliations:** 1 Department of Marine Ecology, Centre for Advanced Studies of Blanes (CEAB, CSIC), Blanes, Spain; 2 Norwegian College of Fishery Science, UiT the Arctic University of Norway, Tromsø, Norway; 3 Department of Evolutionary Biology, Ecology and Environmental Sciences, and Biodiversity Research Institute (IRBio), University of Barcelona, Barcelona, Spain

**Keywords:** Biodiversity assessment, *Cytochrome c oxidase I*, Eukaryotic communities, Marine benthic ecosystems, Metabarcoding pipelines, Ribosomal RNA 18S

## Abstract

Biodiversity assessment of marine hard-bottom communities is hindered by the high diversity and size-ranges of the organisms present. We developed a DNA metabarcoding protocol for biodiversity characterization of structurally complex natural marine hard-bottom communities. We used two molecular markers: the “Leray fragment” of mitochondrial *cytochrome c oxidase* (COI), for which a novel primer set was developed, and the V7 region of the nuclear small subunit ribosomal RNA (18S). Eight different shallow marine littoral communities from two National Parks in Spain (one in the Atlantic Ocean and another in the Mediterranean Sea) were studied. Samples were sieved into three size fractions from where DNA was extracted separately. Bayesian clustering was used for delimiting molecular operational taxonomic units (MOTUs) and custom reference databases were constructed for taxonomic assignment. Despite applying stringent filters, we found high values for MOTU richness (2,510 and 9,679 MOTUs with 18S and COI, respectively), suggesting that these communities host a large amount of yet undescribed eukaryotic biodiversity. Significant gaps are still found in sequence reference databases, which currently prevent the complete taxonomic assignment of the detected sequences. In our dataset, 85% of 18S MOTUs and 64% of COI MOTUs could be identified to phylum or lower taxonomic level. Nevertheless, those unassigned were mostly rare MOTUs with low numbers of reads, and assigned MOTUs comprised over 90% of the total sequence reads. The identification rate might be significantly improved in the future, as reference databases are further completed. Our results show that marine metabarcoding, currently applied mostly to plankton or sediments, can be adapted to structurally complex hard bottom samples. Thus, eukaryotic metabarcoding emerges as a robust, fast, objective and affordable method to comprehensively characterize the diversity of marine benthic communities dominated by macroscopic seaweeds and colonial or modular sessile metazoans. The 18S marker lacks species-level resolution and thus cannot be recommended to assess the detailed taxonomic composition of these communities. Our new universal primers for COI can potentially be used for biodiversity assessment with high taxonomic resolution in a wide array of marine, terrestrial or freshwater eukaryotic communities.

## Introduction

Reliable methods for accurately and objectively assessing the biodiversity of marine environments are needed for a good understanding of these key ecosystems (Costello et al., 2010) and to establish biodiversity baselines and monitor long-term biodiversity changes (Knowlton & Jackson, 2008). Among marine ecosystems, shallow benthic hard-bottom communities are frequently considered to support the highest values of diversity, being arguably the most diverse ecosystems in the biosphere (Reaka-Kudla, 1997; Agardy et al., 2005). Their proximity to humans places them among the best studied and most heavily impacted of all marine biomes. They are also the most influential for human ecology and economy. However, marine ecologists still lack robust, validated and standardized tools for comprehensively surveying these communities.

An exhaustive analysis of these biomes by traditional morphological methods is problematic due to their high complexity, the presence of colonial or modular species, and the abundance of tiny epibiotic forms (Mikkelsen & Cracraft, 2001; Wangensteen & Turon, 2017). In most instances, morphological surveys are limited to macro-organisms, and are often focused on a few taxonomic groups, strongly conditioned by the availability of taxonomic expertise. The taxonomic impediment (Wheeler, Raven & Wilson, 2004) and the occurrence of cryptic species complexes (Knowlton, 1993) further hinder the practicability of morphology-based methods.

In the last few years, the development of metabarcoding techniques, whereby a barcoding marker of the species present in a given sample can be detected by high-throughput sequencing and identified using molecular databases (Hajibabaei et al., 2011; Taberlet et al., 2012), has revolutionized biodiversity assessment. Metabarcoding approaches have been successfully used to characterize marine communities in relatively homogeneous substrates such as seawater (e.g.,  De Vargas et al., 2015; Chain et al., 2016) or marine sediments (e.g., Chariton et al., 2010; Fonseca et al., 2014; Pawlowski et al., 2014; Guardiola et al., 2015; Lejzerowicz et al., 2015) containing mostly small-sized organisms. Leray & Knowlton (2015) introduced methods for analysing the community DNA extracted from organisms collected in autonomous reef monitoring structures (ARMS) using COI metabarcoding. These artificial-substrate communities have also been analysed using other markers such as the 18S gene (Pearman et al., 2016). However, metabarcoding methods have not been used to characterize complex communities dwelling on marine natural hard-bottom substrates. These environments pose new challenges related to sample treatment (given the orders-of-magnitude variation in organisms’ sizes) and to the need of amplifying the wide array of taxonomic groups inhabiting these communities.

In the present work, we introduce a metabarcoding protocol for characterizing complex communities inhabiting natural marine hard substrates. The suitability and robustness of our methods are assessed by comparing the results from two independent universal eukaryotic molecular markers: a fragment of the nuclear gene for the small subunit of the ribosomal RNA (18S) and a fragment of the *cytochrome c oxidase subunit I* mitochondrial gene (COI). A multigene metabarcoding approach has been advocated to overcome limitations inherent to single marker studies (Drummond et al., 2015; Clarke et al., 2017; Kelly et al., 2017). The COI and 18S genes generally provide coherent results in terms of taxonomic results and *β*-diversity (Drummond et al., 2015; Kelly et al., 2017; Clarke et al., 2017), although the former results in higher number of MOTUs identified to the species level (Cowart et al., 2015) and enhanced taxonomic resolution (Tang et al., 2012). On the other hand, more MOTUs remain unassigned with COI (Cowart et al., 2015), and most primer sets used for COI amplification are not universal for eukaryotes and may fail to amplify some taxa (Deagle et al., 2014). We have modified an existing primer set by increasing degeneracy to improve universality in the amplification of the “Leray fragment” (Leray et al., 2013) of COI in most eukaryotic groups.

Size fractionation of bulk samples has been proposed as a convenient step in metabarcoding when organisms in a sample have unequal biomass (Elbrecht, Peinert & Leese, 2017). This procedure has been used in the marine environment for metabarcoding macrobenthos in sediment samples (e.g., Aylagas et al., 2016), mobile and sessile organisms on settlement plates (e.g., Leray & Knowlton, 2015; Ransome et al., 2017), or zooplankton (e.g., Liu et al., 2017), but it has never been applied to samples from natural hard-bottom communities, with a high inherent complexity and organisms spanning several orders of magnitude in size. We sieved each sample into three size fractions, corresponding to the distinction between mega-, macro- and meiobenthos (Rex & Ettter, 2010), which has important implications in terms of structure and function of benthic communities (e.g., Warwick & Joint, 1987; Galéron et al., 2000; Rex et al., 2006).

This case study focused on eight shallow benthic communities sampled within Marine Protected Areas (MPAs) at two distinct Spanish National Parks. They constitute a convenient setting for this study, as management of MPAs requires efficient biomonitoring over time. Our main objective was to develop and apply a method for characterizing complex marine hard-substrate communities using community DNA metabarcoding. To this end, we (1) developed adapted field sampling protocols, (2) tested the effects of size-fractionation in the detection of marine taxa spanning a wide range of sizes, (3) improved existing resources by testing a modified primer set for COI and generating new reference databases, (4) compared the relative performance of 18S and COI markers in terms of taxonomic accuracy and biodiversity patterns. In addition, our research sought to generate baseline information for biodiversity assessment and biomonitoring of these benthic communities.

## Materials and Methods

### Sampling

Samples were taken by scuba diving from eight shallow hard-bottom communities inside two national parks in Spain: Cíes Islands (Atlantic Islands National Park, Galicia, Northeastern Atlantic) and Cabrera Archipelago National Park (Balearic Islands, Western Mediterranean). A map of sampling locations is shown in Fig. S1. The rationale for the choice of the communities was to have the most representative habitats along a depth gradient of the rocky littoral of these national parks for the purpose of obtaining baseline inventories for future monitoring and management efforts. Atlantic communities were sampled in May 2014, and the Mediterranean ones in September 2014. Table 1 lists the main characteristics of the studied assemblages, while Fig. S2 shows representative images of them. In short, two photophilous communities and one sciaphilous community were sampled at both parks (note that light levels are higher in the clear Mediterranean waters, hence equivalent communities are placed deeper than in the Atlantic, Table 1). In addition, detritic rhodolith beds (also known as maërl bottoms, Peña & Bárbara, 2008) were also sampled. Although these detritic bottoms are not strictly rocky communities, they are included in this work because they share the three-dimensional complexity (Peña & Bárbara, 2008; Joher, Ballesteros & Rodríguez-Prieto, 2016) and much of the biodiversity present, as we sampled communities just adjacent to rocky slopes. We will hereafter name the communities after the dominant species, except for the sciaphilous community in Cabrera (hereafter “precoralligenous” community), which did not have a clearly landscape-dominant species, and the two detritic communities (hereafter detritic Atlantic and detritic Mediterranean), for the same reason.

**Table 1:** Characteristics of the eight communities studied at the two National Parks. The sampling method used is also indicated.

Ocean basin	Light level	Community	Dominant species	Depth (m)	Coordinates	Sampling method
Mediterranean	high	Photophilous algae	*Lophocladia lallemandii*	7–10	39.1253,2.9604	quadrats (25*25 cm)
Mediterranean	high	Photophilous algae	*Padina pavonica*	7–10	39.1251,2.9603	quadrats (25*25 cm)
Mediterranean	low	Sciaphilous algae	Sponges and other invertebrates	30	39.1250,2.9602	quadrats (25*25 cm)
Mediterranean	low	Detritic bottom	Red coralline algae	65	39.1249,2.9604	corers (30 cm ø* 5 cm)
Atlantic	high	Photophilous algae	*Cystoseira nodicaulis*	3–5	42.2259,−8.8969	quadrats (25*25 cm)
Atlantic	high	Photophilous algae	*Cystoseira tamariscifolia*	3–5	42.2260,−8.8970	quadrats (25*25 cm)
Atlantic	low	Sciaphilous algae	*Saccorhiza polyschides*	16	42.1917,−8.8885	quadrats (25*25 cm)
Atlantic	low	Detritic bottom	Red coralline algae	20	42.2123,−8.8972	corers (30 cm ø* 5 cm)

All rocky-bottom communities (three replicates each) were sampled by carefully scraping a 25 × 25 cm quadrat with chisel and hammer (Fig. S2). The detritic communities were sampled (three replicates each) by using a cylindrical PVC corer of equivalent area (24 cm in diameter) and sampling the first 5 cm of the community (Fig. S2). All samples were placed underwater inside polyethylene bags. Water was eliminated through a 63 µm mesh sieve shortly after sampling, being then replaced by 96% ethanol. The material retained in the filter was washed back to the sample bag with ethanol. Samples were stored at −20 °C until further processing.

### Sample pre-treatment, controls, DNA extraction and reproducibility tests

The samples were separated into three size fractions (A: >10 mm; B: 1–10 mm; C: 63 µm–1 mm) (mega-, macro- and meiobenthos, Rex & Ettter, 2010) using a column of stainless steel sieves (CISA Cedaceria Industrial S.A., Barcelona, Spain), washing thoroughly under high-pressure freshwater. All separated fractions were then recovered in 96% ethanol, homogenized using a 600 W kitchen blender and stored at −20 °C until DNA extraction. All equipment was thoroughly washed and cleaned with diluted sodium hypochlorite between successive samples. Two negative controls for the pre-treatment separation protocol were done by using sand samples charred in a muffle furnace at 400 °C for 24 h to remove all traces of DNA. This muffled sand was sieved and extracted using the same procedure used for the samples.

For total DNA extraction, 10 g of each homogenized sample were purified using PowerMax Soil DNA Isolation Kit (QIAGEN, Valencia, CA, USA). DNA concentration of extracts was assessed in a Qubit fluorometer (Life Technologies, Carlsbad, CA, USA) using a high sensitivity assay and, if needed, concentrated in a Savant DNA120 Speedvac system (Thermo-Scientific, Waltham, MA, USA) until DNA concentration of >5 ng/µl was achieved. Two PCR-blanks were run by amplifying the elution buffer of the DNA isolation kit as template.

Technical replicates were used to empirically test data reproducibility (Leray & Knowlton, 2017). One of the homogenized samples (from the atlantic community dominated by *Cystoseira tamariscifolia*) was extracted in triplicate and amplified independently, in order to check the reproducibility of the DNA extraction procedure. One of these extractions was then amplified using three PCR reactions with different sample tags, in order to check the reproducibility of the PCR amplification and to confirm the absence of bias introduced by mismatches due to sample tags (O’Donnell et al., 2016). The variability of these samples (due to biases and errors during the PCR in the latter case, plus the variability in the DNA extraction procedure in the former case) was also compared with the natural ecological variability, that was assessed by the three different replicates obtained from the same community.

### DNA amplification and library preparation

Two different metabarcoding markers were amplified: 18S and COI. For the V7 region of 18S rRNA, the recently developed 18S_allshorts primers were used: forward: 5′-TTTGTCTGSTTAATTSCG-3′ and reverse: 5′-TCACAGACCTGTTATTGC-3 amplifying a ca. 110 bp fragment (Guardiola et al., 2015). These primers show a high universality across eukaryotic groups (see *in silico* analysis and primer logos in Guardiola et al., 2015). To these primers, 8-base sample-specific tags were attached. We used the same tag at both primers in order to detect inter-sample chimeric sequences. Moreover, a variable number (2–4) of fully degenerated positions (Ns) were added to the 5′ end to enhance molecular diversity during sequencing. The PCR conditions followed Guardiola et al. (2015), using a standardized amount of 10 ng of purified DNA per sample.

For COI, we amplified the “Leray fragment” of ca. 313 bp using a new highly degenerated primer set (henceforth Leray-XT). This set included the reverse primer jgHCO2198 5′-TAIACYTCIGGRTGICCRAARAAYCA-3′ (Geller et al., 2013) and a novel forward primer mlCOIintF-XT 5′-GGWACWRGWTGRACWITITAYCCYCC-3′, modified from the mlCOIintF primer (Leray et al., 2013) by incorporating two more degenerate bases and two inosine nucleotides in the most variable positions. This was done after manually checking the original primer against representative sequences of the main eukaryotic groups obtained from the Genbank database. Sample tags and fully degenerate positions were attached to both primers as before. Additionally, we performed an *in silico* analysis to compare the coverage of the new primer set for COI with the original Leray set (Leray et al., 2013), which is included as File S1. More detailed tests to assess the performance of the new Leray-XT primer set on artificial mock communities are currently underway but fall beyond the scope of this work and will be published elsewhere.

Amplification of COI used AmpliTaq Gold DNA polymerase (Applied Biosystems, Foster City, CA, USA), with 1 µl of each 5 µM forward and reverse 8-base tagged primers, 3 µg of bovine serum albumin and 10 ng of purified DNA in a total volume of 20 µl per sample. The PCR profile included 10 min at 95 °C, 35 cycles of 94 °C 1 min, 45 °C 1 min and 72 °C 1 min, and 5 min at 72 °C.

After PCR, quality of amplifications was assessed by electrophoresis in agarose gels. All PCR products were purified using Minelute PCR purification columns (Qiagen, Valencia, CA, USA) and pooled by marker. Two Illumina libraries were built from the DNA pools using the Metafast protocol at Fasteris SA (Plan-les-Ouates, Switzerland). This protocol incorporates Illumina adapters using a ligation procedure without any further PCR step, thus minimising biases. Each library was sequenced independently in an Illumina MiSeq platform using v3 chemistry (2 ×150 bp paired-end run for 18S and 2 ×300 bp paired-end run for COI).

### Metabarcoding pipeline

We based our metabarcoding pipeline on the OBITools v1.01.22 software suite (Boyer et al., 2016). The length of the raw reads was trimmed to a median Phred quality score higher than 30, after which paired-reads were assembled using illuminapairedend. The reads with paired-end alignment quality scores higher than 40 were demultiplexed using ngsfilter, which also removed the primer sequences. A length filter (obigrep) was applied to the assigned reads (75–180 bp for 18S and 300–320 bp for COI). The reads were then dereplicated (using obiuniq) and chimeric sequences were detected and removed using the uchime_denovo algorithm implemented in vsearch v1.10.1 (Rognes et al., 2016).

The MOTUs were delimited using the Bayesian clustering algorithm implemented in CROP v1.33 (Hao, Jiang & Chen, 2011). This algorithm results in variable thresholds for delimiting MOTUs across different branches of the taxonomic tree, following the natural organization of the clusters in multidimensional sequence space. The following parameter sets were used: *l* = 0.3, *u* = 0.5 for 18S (Guardiola et al., 2016) and *l* = 1.5, *u* = 2.5 for COI. These values for COI are more relaxed than the original values previously used for this fragment (Leray et al., 2013; Leray & Knowlton, 2015) and were chosen to avoid overclustering of several unrelated species into single MOTUs (Wangensteen & Turon, 2017).

The taxonomic assignment of the representative sequences for each MOTU was performed using ecotag (Boyer et al., 2016), which uses a local reference database and a phylogenetic tree-based approach (using the NCBI taxonomy) for assigning sequences without a perfect match. Ecotag searches the best hit in the reference database and builds a set of sequences in the database which are at least as similar to the best hit as the query sequence is. Then, the MOTU is assigned to the most recent common ancestor to all these sequences in the NCBI taxonomy tree. With this procedure, the assigned taxonomic rank varies depending on the similarity of the query sequences and the density of the reference database. For 18S, we used the db_18S_r117 reference database (Guardiola et al., 2015), obtained by *in silico* ecoPCR (Ficetola et al., 2010) with the 18S_allshorts primer set against the release 117 of the EMBL nucleotide database. This database includes 26,125 reference sequences from all major eukaryotic groups. For COI, we developed a mixed reference database by joining sequences obtained from two sources: *in silico* ecoPCR against the release 117 of the EMBL nucleotide database and a second set of sequences obtained from the Barcode of Life Datasystems (Ratnasingham & Hebert, 2007) using a custom R script to select the Leray fragment. This newly generated database (db_COI_MBPK) included 188,929 reference sequences (March 2016). We did not add any new sequences to build our custom reference databases. Instead, we deliberately used only sequences already available from public repositories in order to assess the completeness of current barcoding databases for marine taxa. Both reference databases are publicly available from http://github.com/metabarpark/Reference-databases and a summary of the taxa represented in them is shown in Table S1.

After taxonomic assignment, final refining of the datasets was performed. A control correction was made following Wangensteen & Turon (2017): all MOTUs for which the abundance in the blanks and negative controls was higher than 10% of the total reads of that MOTU were removed. We also eliminated spurious positive results due to random tag switching. To this end, we used the “abundance renormalization” procedure described in Wangensteen & Turon (2017), consisting of sorting the samples by abundance of each MOTU and eliminating the reads of the samples corresponding to a cumulative frequency of less than 3% for each particular MOTU. This “across samples” filtering was followed by an “across MOTUs” minimal relative abundance filtering, where all MOTUs that did not have a relative abundance greater than 0.01% in at least one sample were removed. Since we were interested only in eukaryotic diversity, all MOTUs assigned to prokaryotes or to the root of the Tree of Life were removed from the analyses. Samples having less than 10,000 reads in the final datasets, after all filtering procedures, were considered as failed and deleted from the analyses.

The details of the pipelines used for both metabarcoding markers are listed in Table S2. Note that, rather than following exactly the same pipeline for both markers, we adapted some steps to the particularities of each gene to obtain and compare the best information that can be gleaned from each. For instance, singleton sequences (3.4% of reads) were removed before clustering into MOTUs in the 18S pipeline, as is commonly done. However, this is not recommended in the case of COI, since singletons made up a large proportion of reads (30.15%) in this long variable marker. These singletons were mostly single point variants that would be included in the correct MOTU during clustering. Early removal of singletons could lead to an excessively pruned dataset for long markers (Wangensteen & Turon, 2017), thereby decreasing notably the number of final reads per sample. Truly divergent singletons were removed along with all MOTUs with less than five reads after the clustering step in our COI pipeline. Likewise, parameters of the CROP procedure were tailored to the characteristics of both markers (see above). Finally, merging of MOTUs assigned to the same species was performed for COI but not for 18S, as different 18S sequences in this marker almost invariably correspond to different species that were assigned to the same species-level taxon because of gaps in the reference databases. The original sequences, after pairing and quality checks, are available from the Mendeley data repository (https://data.mendeley.com/datasets/nm2c97fjng).

### Statistical analyses

Analyses of *α*-diversity were carried out using Primer v6 (Clarke, 1993) and two metrics: species richness and the Shannon index. For the former, we used a rarefaction size of 19,000, determined by the sample containing less reads (19,819), PERMANOVA v1 (Anderson, Gorley & Clarke, 2008) was used to formally analyse the effect of site (Atlantic or Mediterranean), community (nested within site), and fraction on *α*-diversity metrics. Sample replicate was used as a blocking factor within community and 1,000 permutations were performed using Euclidean distances to test for the different effects. *A posteriori* pairwise tests were performed with PERMANOVA for the main factor fraction whenever significant. When the community factor was significant, the pairwise tests of this factor were made within each site.

The rest of the analyses were performed in R 3.3.0 (R Core Team, 2016). Package vegan (Oksanen et al., 2016) was used for calculations of Bray–Curtis dissimilarity matrices (function vegdist), comparison of these matrices (function mantel), and group representation in non-metric multidimensional scaling (nmMDS) diagrams (function metaMDS). All calculations of Bray–Curtis dissimilarities were performed using fourth square root-transformed values of relative frequencies of read numbers of MOTUs in each sample.

For assessing the reproducibility of the extraction and PCR procedures, Bray–Curtis distances were calculated as above between technical replicates and compared with distances obtained from ecological replicates. For assessing the effect of size fractionation on the detectability of organisms with different sizes, we obtained the percentages of reads for each of the following categories of MOTUs: (1) macroscopic seaweeds, (2) modular metazoa, (3) macrofaunal unitary metazoa, (4) meiofaunal metazoa, (5) microorganisms and (6) unassigned.

## Results

### Sequencing depth and *α*-diversity patterns

We metabarcoded a total of 80 samples per marker (36 subsamples from four benthic communities in Cíes, 36 from four communites in Cabrera, four additional samples used for studying reproducibility, two blanks and two negative controls). One sample from the 18S dataset and four samples from the COI dataset were removed from the analyses due to low number of reads (<10,000). Controls had a negligible number of reads (average 65 reads, maximum 204 reads). After all filtering procedures, our final dataset for 18S comprised a total of 7,640,737 reads, with an average of 107,616 reads per sample (range: 62,583–189,036). For COI, our final dataset included 9,128,836 reads, with an average of 134,248 reads per sample (range: 19,819–417,729).

The number of total MOTUs detected from all samples by Bayesian clustering was 2,510 for 18S, from which 2,141 (85,3%) could be assigned to the level of phylum or lower. These assigned MOTUs accounted for 97.2% of the total 18S reads. The number of MOTUs yielded by COI from the same samples was higher: 9,679, from which 6,145 (63,5%) could be taxonomically assigned to the level of phylum or lower. The assigned MOTUs accounted for 92.1% of total COI reads. Our final datasets, including representative sequences, taxonomic assignment, and abundance of all MOTUs, are presented in Tables S3 and S4.

**Figure 1:**
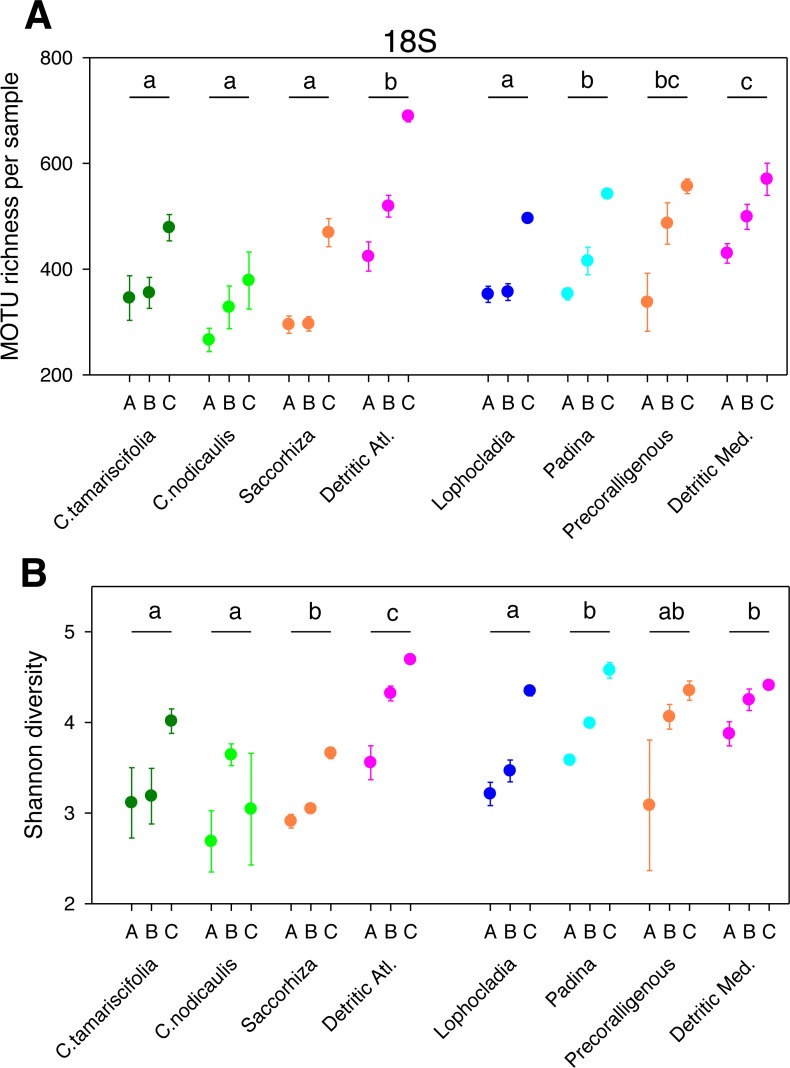
Scatter plots showing patterns of MOTU richness (A) and Shannon diversity index (B) for the 18S gene. Results from eight different sublittoral communities (means ±  SE shown). Fraction A, coarse; B, intermediate; C, fine. MOTU richness results obtained by rarefaction analysis to 19,000 reads per sample. Whenever a significant community effect was found in the PERMANOVA analyses (Table 2), the letters above the communities represent groups not significantly different in a *post-hoc* pairwise test within each National Park.

**Figure 2:**
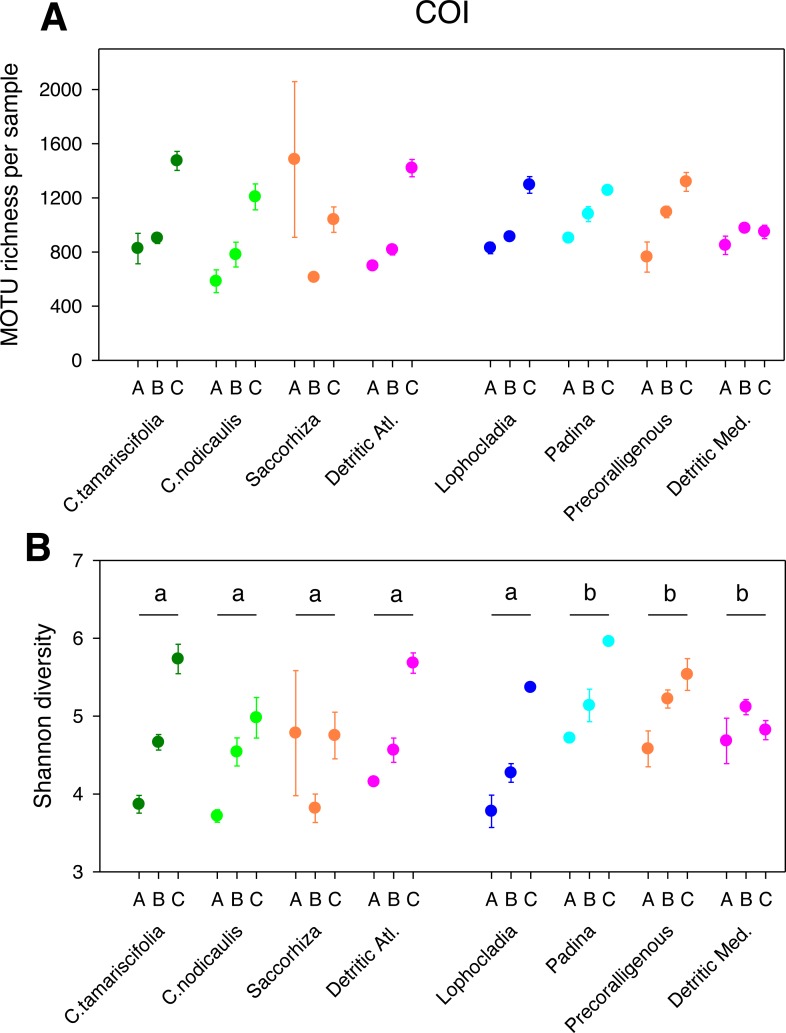
Scatter plots showing patterns of MOTU richness (A) and Shannon diversity index (B) for the COI gene. Results from eight different sublittoral communities (means ±  SE shown). Fraction A, coarse; B, intermediate; C, fine. MOTU richness results obtained by rarefaction analysis to 19,000 reads per sample. Whenever a significant community effect was found in the PERMANOVA analyses (Table 2), the letters above the communities represent groups not significantly different in a *post-hoc* pairwise test within each National Park.

The different fractions of the sampled communities showed the same patterns of MOTU richness and diversity for 18S and COI after rarefaction (Figs. 1 and 2). Using either marker, a trend can be observed whereby richness and diversity increased from the coarser to the finer fraction in most communities. Only in the Mediterranean detritic community with COI and the *Cystoseira nodicaulis* community with 18S was the fraction C less diverse than B. Another exception was the high mean value of diversity of the fraction A of the *Saccorhiza* community with COI, with a particularly large dispersion among replicates. Fraction A of the *C. nodicaulis* community had the lowest values of (rarefied) MOTU richness per sample for both genes (266.07 ± 15.6 for 18S and 583.17 ± 48.5 for COI, mean ± SE), while fraction C of the atlantic detritic community had the highest values (689.41 ±7.7 MOTUs/sample for 18S and 1418.85 ± 45.1 for COI). The same communities had the lowest and highest, respectively, values of the Shannon diversity index (ranging from 2.69 ± 0.24 to 4.58  ± 0.14 for 18S and from 3.72 ± 0.04 to 5.68 ± 0.09 for COI).

The PERMANOVA analyses (Table 2) showed that for both *α*-diversity metrics and markers, the effect of site (Atlantic or Mediterranean) was not significant, while fraction had highly significant effects. The community factor was significant except for the COI comparison of richness. The replicate (sample) factor was not significant except for the 18S values of Shannon diversity. Pairwise tests for the overall effect of fraction showed a common trend of higher diversity in fraction C, but not all pairwise comparisons were significant in all cases (Table 2). Likewise, there were significant differences in pairwise tests between communities that varied according to the gene and the metric considered (Figs. 1 and 2), but overall the results for 18S reflected a higher richness and diversity in the sciaphilous and detritic communities, while for COI the only significant difference was between the *Lophocladia* community and the rest of Mediterranean communities.

**Figure 3:**
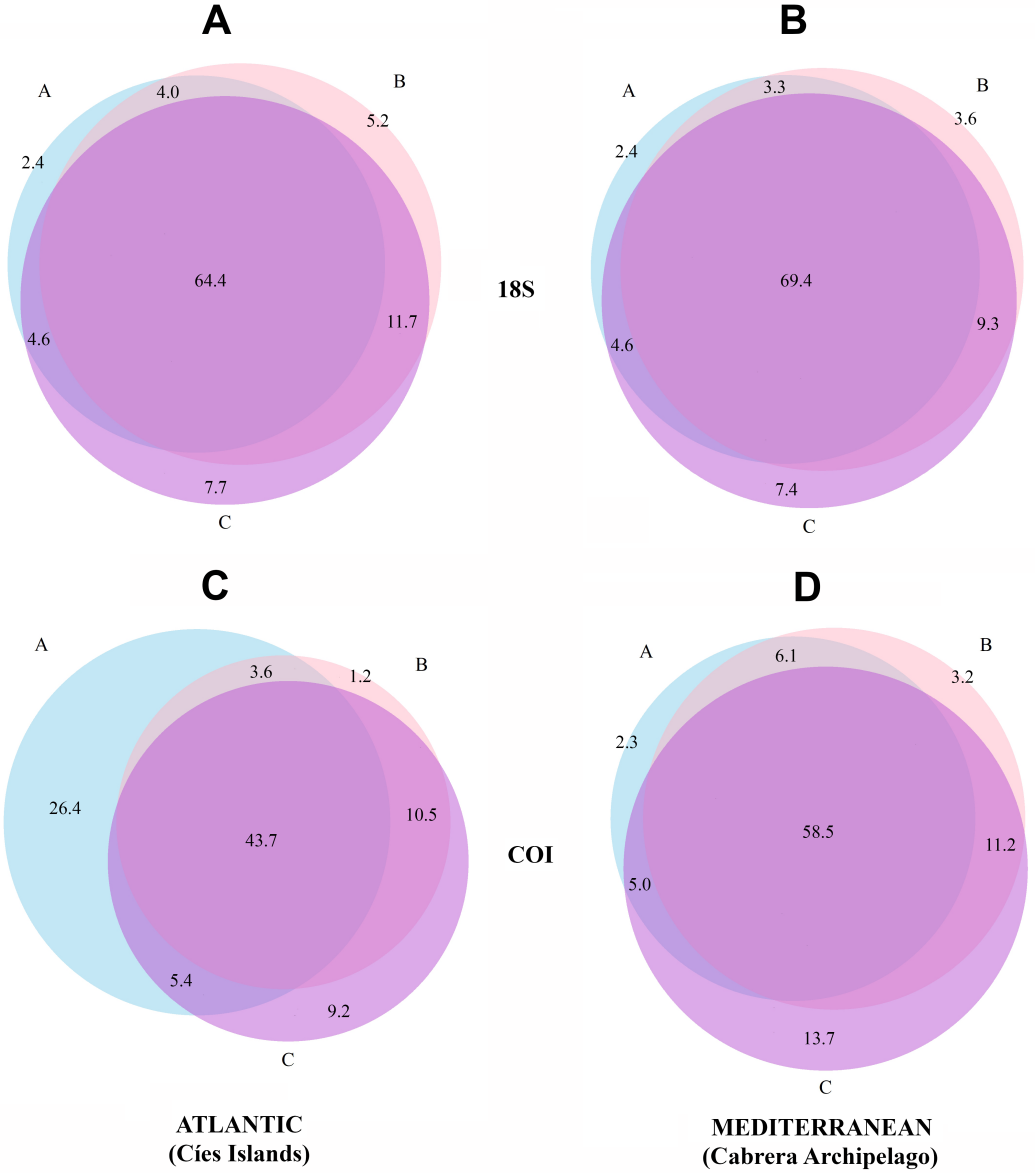
Venn diagrams showing the number of MOTUs detected in each size fraction. Results are presented separately for each gene and for the Atlantic and Mediterranean National Parks. (A) 18S in the Atlantic, (B) 18S in the Mediterranean, (C) COI in the Atlantic, (D) COI in the Mediterranean. Numbers are percentages of total MOTUs. Fraction A, coarse; B, intermediate; C, fine.

Venn diagrams representing the MOTUs detected in the three fractions are presented separately for the two National Parks in Fig. 3. There is a higher overlap when using 18S (69.4% and 64.4% of MOTUs were detected in the three fractions in Cabrera and Cíes Islands, respectively), while this overlap was substantially reduced with COI (58.5% and 43.7% of MOTUs, respectively). In addition, fraction C of COI had between 9.2% (Cies) and 13.7% (Cabrera) of exclusive MOTUs, while these percentages were just 7.7% and 7.4%, respectively, in fraction C of 18S. Between ca. 20% and ca. 28% of MOTUs, depending on the gene and National Park considered, were found exclusively in the two smaller fractions (B and C).

**Table 2:** Results of the permutational analysis of variance (PERMANOVA) of the rarefied MOTU richness and Shannon diversity values for the two genes studied. *Post-hoc* pairwise tests for the significant main factor fraction are indicated. Pairwise tests for the community factor, when significant, are presented graphically in Figs. 1 and 2.

	*df*	SS	MS	Pseudo-*F*	*p*-value	post-hoc test
**18S MOTU richness**						
Site	1	3.57E+04	3.57E+04	0.72305	0.432	
Fraction	2	3.54E+05	1.77E+05	43.712	0.001	A < B < C
Site*fraction	2	3,411.1	1,705.6	0.42163	0.634	
Community (site)	6	2.97E+05	49,539	15.711	0.001	
Sample (community)	16	50,559	3,160	1.6166	0.121	
Residual	31	60,594	1,954.6			
**18S Shannon diversity**						
Site	1	3.421	3.421	2.7364	0.134	
Fraction	2	8.9238	4.4619	17.815	0.002	A < B = C
Site*fraction	2	0.13855	6.93E−02	0.27659	0.765	
Community (site)	6	3.0166	0.25138	2.154	0.045	
Sample (community)	16	5.078	0.31738	2.7195	0.007	
Residual	31	3.6178	0.1167			
**COI MOTU richness**						
Site	1	18,869	18,869	0.41923	0.523	
Fraction	2	1.70E+06	8.51E+05	5.78E+00	0.026	A = B < C
Site*fraction	2	3.39E+05	1.70E+05	1.153	0.336	
Community (site)	6	2.65E+05	4.42E+04	1.0125	0.495	
Sample (community)	16	6.84E+05	4.28E+04	0.66873	0.864	
Residual	28	1.79E+06	6.39E+04			
**COI Shannon diversity**						
Site	1	1.6876	1.6876	2.7634	0.117	
Fraction	2	11.172	5.5859	11.034	0.004	A = B < C
Site*fraction	2	0.35745	0.17872	0.35305	0.702	
Community (site)	6	3.7634	0.62724	5.1615	0.004	
Sample (community)	16	1.881	0.11756	0.56391	0.892	
Residual	28	5.8373	0.20847			

### Taxonomic assignment and database gaps

The number of sequences from different taxonomic groups included in the reference databases used for our analyses is summarized in Table S1. Although the total number of different reference sequences for COI is one order of magnitude higher than for 18S, some important taxonomic groups are remarkably absent from the COI reference database, such as Choanozoa, Foraminifera or several fungal phyla. Among groups with macro-organisms, the low representation of Viridiplantae in the COI database is noteworthy, while Chordata are poorly represented in the 18S database (1.3% of the total sequences vs 21.2% in COI).

**Figure 4:**
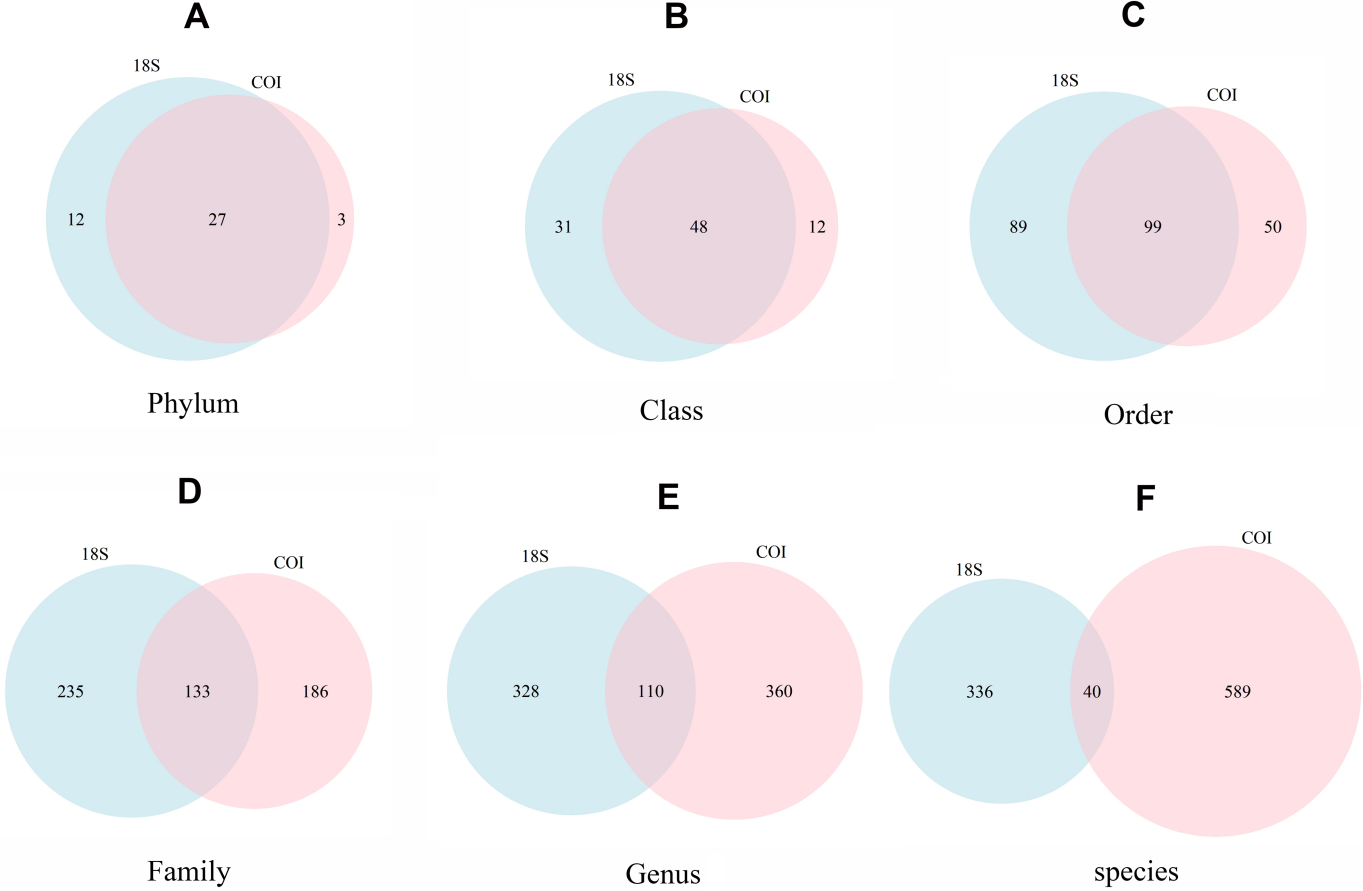
Venn diagrams showing the number of different taxa recovered with 18S (blue) or COI (red) at different taxonomic ranks. (A) Phylum, (B) Class, (C) Order, (D) Family, (E) Genus, (F) species.

The number of taxa identified at phylum or lower categories for both markers is shown in Fig. 4. A clear trend emerges: the lower the category, the less coincidence between the taxa found with both markers. Thus, at the phylum level, 90.0% of the phyla detected with COI were also recovered with 18S. The corresponding figures were 80.0% for class, 66.4% for order, 41.7% for family, 23.4% for genus, and 6.4% for species-level taxa. Moreover, 18S detected a higher number of taxa in the higher categories, but lower for genus and, particularly, species level (629 species were identified with COI vs only 376 with 18S).

**Figure 5:**
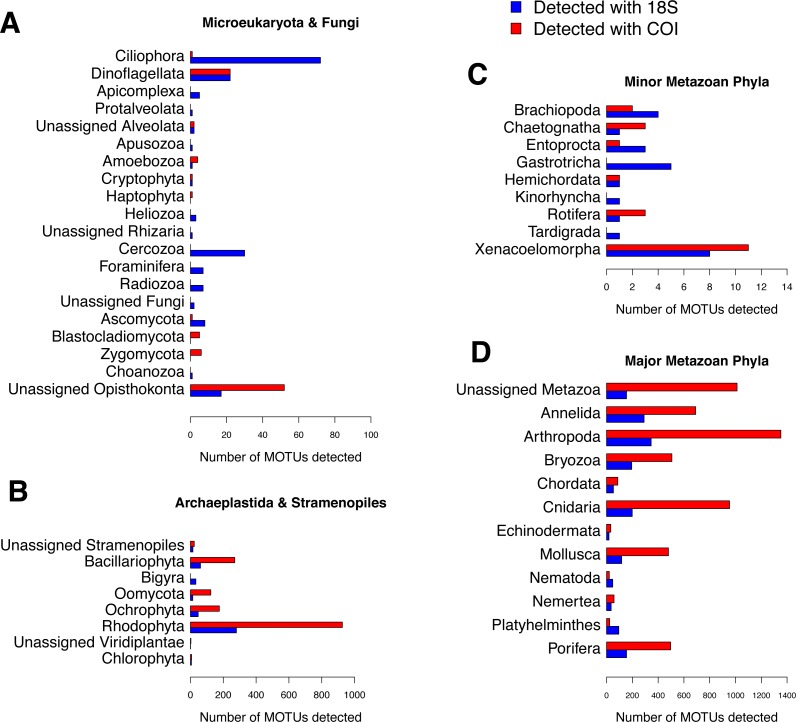
Number of MOTUs detected for every phylum in the studied communities using 18S (blue) or COI (red). Note the different scales used for (A) microeukaryota and Fungi, (B) Archaeplastida and Stramenopiles, (C) minor metazoan phyla, and (D) major metazoan phyla.

The numbers of MOTUs detected in the samples by phylum (Fig. 5) showed that both markers, COI and 18S, were able to detect those groups composed of medium- or big-sized organisms, such as major metazoan phyla or macroscopic seaweeds. The detection of groups comprising microscopic organisms was usually more reliable using 18S than COI. For example, 17 metazoan phyla could be detected in our samples using COI, while the 18S assignment detected these same 17 phyla plus the microscopic Kinorhyncha, Gastrotricha and Tardigrada. Due to remarkable gaps in the reference database (as seen in Table S1), our assignment procedure for COI was unable to identify any sequence from microscopic groups such as Apicomplexa, Apusozoa, Choanozoa, Heliozoa, Protalveolata or Rhizaria (including Foraminifera, Cercozoa and Radiozoa), which could be detected by 18S. However, COI was able to detect and distinguish a higher number of MOTUs than 18S for most macroscopic phyla. The pattern of abundances of MOTU identified at the different taxonomical levels shows that, in general, high-abundance MOTUs could be identified to the species level using COI, whereas they were often identified to higher taxonomic ranks using 18S (Fig. S3). Unassigned MOTUs are those with the least abundances, using either marker (Fig. S3).

### Patterns of MOTU abundance and *β*-diversity

**Figure 6:**
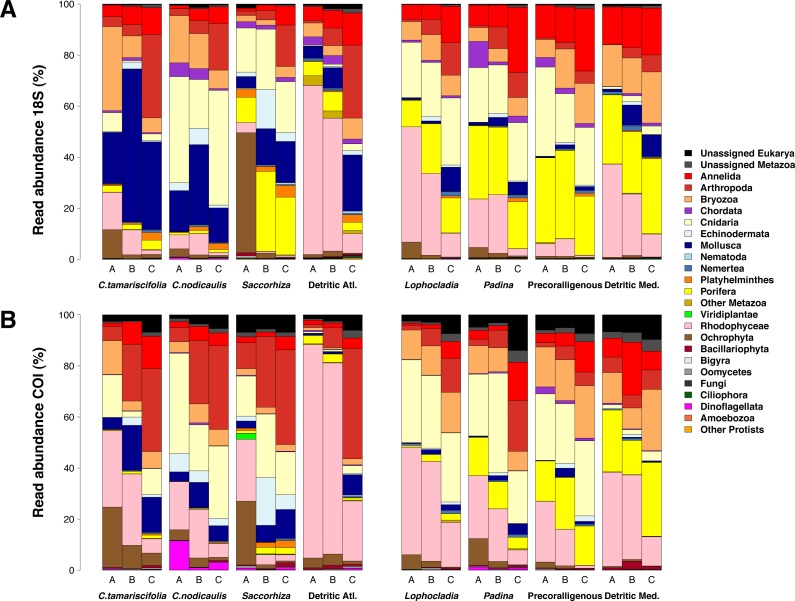
Patterns of relative read abundance per community and fraction size. Results obtained using COI (A) or 18S (B) in eight different marine littoral communities from the Atlantic and Mediterranean National Parks studied. All replicates from the same community and fraction size have been pooled. Fraction A, coarse; B, intermediate; C, fine. See Fig. S4 for the same figure, split by replicates.

The relative read abundances of major eukaryotic groups are presented in Fig. 6 for COI and 18S (the same information split by replicate samples is presented in Fig. S4). The rates of unassigned sequences were, in all cases, higher for COI than for 18S. The unassigned reads were in general most abundant in the smallest fraction (fraction C) of each community. Sequences identified as small Metazoa such as Annelida or Arthropoda were also clearly more abundant in the smallest fractions, whereas big macroscopic seaweeds such as Rhodophyta or Phaeophyceae tended to be dominant in the biggest fractions (A and B). Colonial and modular Metazoa such as Porifera, Cnidaria or Bryozoa were distributed across all fraction sizes. Although some differences may be observed between both markers (e.g.,: higher abundance of reads of Mollusca and Porifera from 18S and more reads of Arthropoda and Rhodophyta from COI), the overall patterns of read abundances were similar for 18S and COI. The three ecological replicates per community were also similar in composition (Fig. S4).

**Figure 7:**
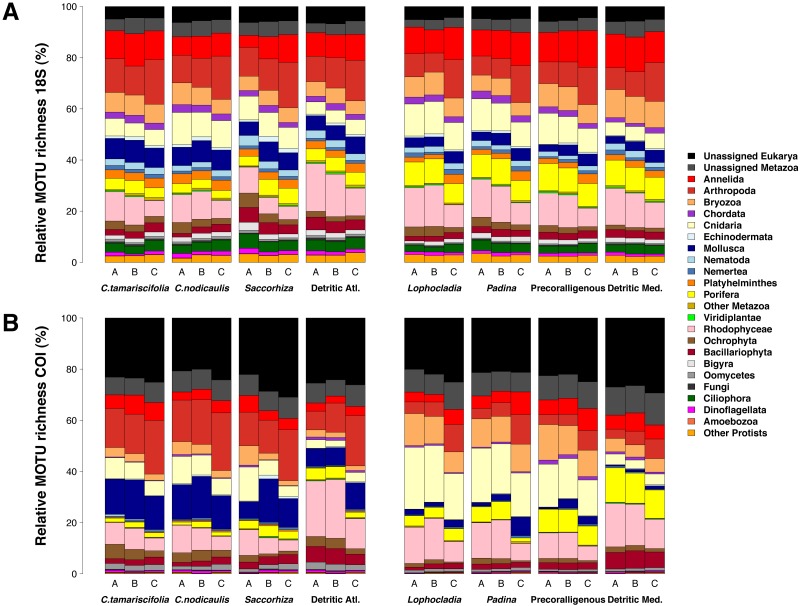
Patterns of relative MOTU richness per community and fraction size. Results obtained using COI (A) or 18S (B) in eight different marine littoral communities from the Atlantic and Mediterranean National Parks studied. All replicates from the same community and fraction size have been pooled. Fraction A, coarse; B, intermediate; C, fine. See Fig. S5 for the same figure, split by replicates.

Figure 7 shows the number of MOTUs assigned to the different phyla for COI and 18S. Compared to the read abundances of Fig. 6, a higher percentage of unassigned MOTUs and a higher dominance of phyla comprising small organisms is apparent. The percentages of MOTUs assigned to microeukaryotes were notably higher for 18S than for COI: the sum of MOTUs assigned to ciliates, dinoflagellates, Bigyra and other protists accounted for 11.22% of assigned 18S MOTUs averaged over samples, while this sum was just 3.71% of the assigned COI MOTUs. The same happens with small-sized metazoans, for which the relative number of MOTUs assigned was higher for 18S (e.g., annelids 52% higher, nematodes 10 times higher, flatworms 12 times higher). In contrast, COI detected higher relative number of MOTUs per sample than 18S for large organisms such as rhodophytes (30.5% higher), ochrophytes (17.1% higher), cnidarians (63.7% higher), arthropods (23.8% higher), or mollusks (71.0% higher). Again, the three replicates per community showed a similar composition in terms of MOTU richness per phylum (Fig. S5).

**Figure 8:**
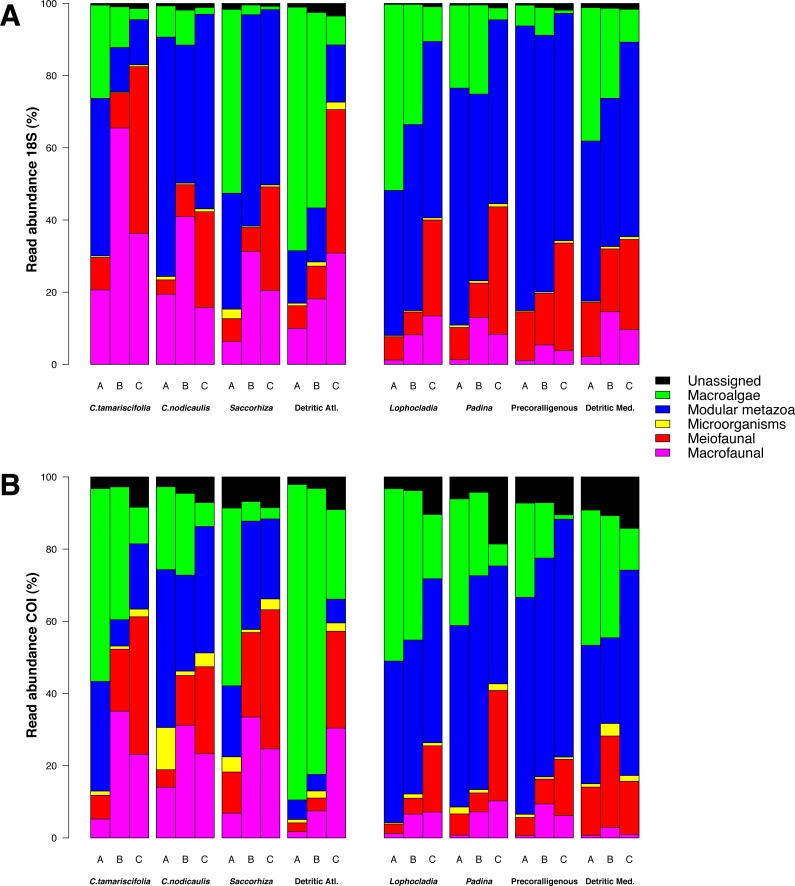
Effect of size fractionation in the recovery of different ecological categories by metabarcoding. Results using COI (A) and 18S (B) on eight different littoral communities from the Atlantic and Mediterranean National Parks studied. All replicates from the same community and fraction size have been pooled. Fraction A, coarse; B, intermediate; C, fine.

Differences between fractions were more evident in terms of read abundance than presence/absence of MOTUs (compare Figs. 6 and 7). The differences were also evident in the percentages of read abundances belonging to different ecological size-categories in the fractions (Fig. 8). Macroalgae were more abundant in fractions A and B, whereas meiofaunal reads were more abundant in fractions C in general. Reads of modular metazoans were more evenly distributed among the three fractions. Interestingly, most reads of microorganisms detected in fractions A and B of these littoral communities, belonged to *Symbiodinium sp. or Amphidinium sp.*, dinoflagellates that are symbionts of macrofaunal anthozoans.

As for the samples used in the replicability analyses, pie charts representing the read abundances of major groups detected at the different levels of replication for both markers are shown in Fig. S6, which highlights the differences in the relative abundances of MOTUs among ecological replicates compared to extraction replicates and PCR replicates, with both markers. Bray–Curtis distances for 18S between three different extractions of the same samples were 0.241 ± 0.008 (mean ± SE), while between three PCR replicates of the same extraction were 0.184 ± 0.005. The values for COI were 0.190 ± 0.002 for extraction replicates and 0.258 ± 0.039 for PCR replicates. These distances were in all cases smaller than the ones found between the three samples (ecological replicates) collected in the same community (0.438  ± 0.028 for 18S, 0.396 ± 0.020 for COI). Thus, differentiation between technical replicates was on average half the one found between ecological replicates, indicating a good reproducibility of the method.

**Figure 9:**
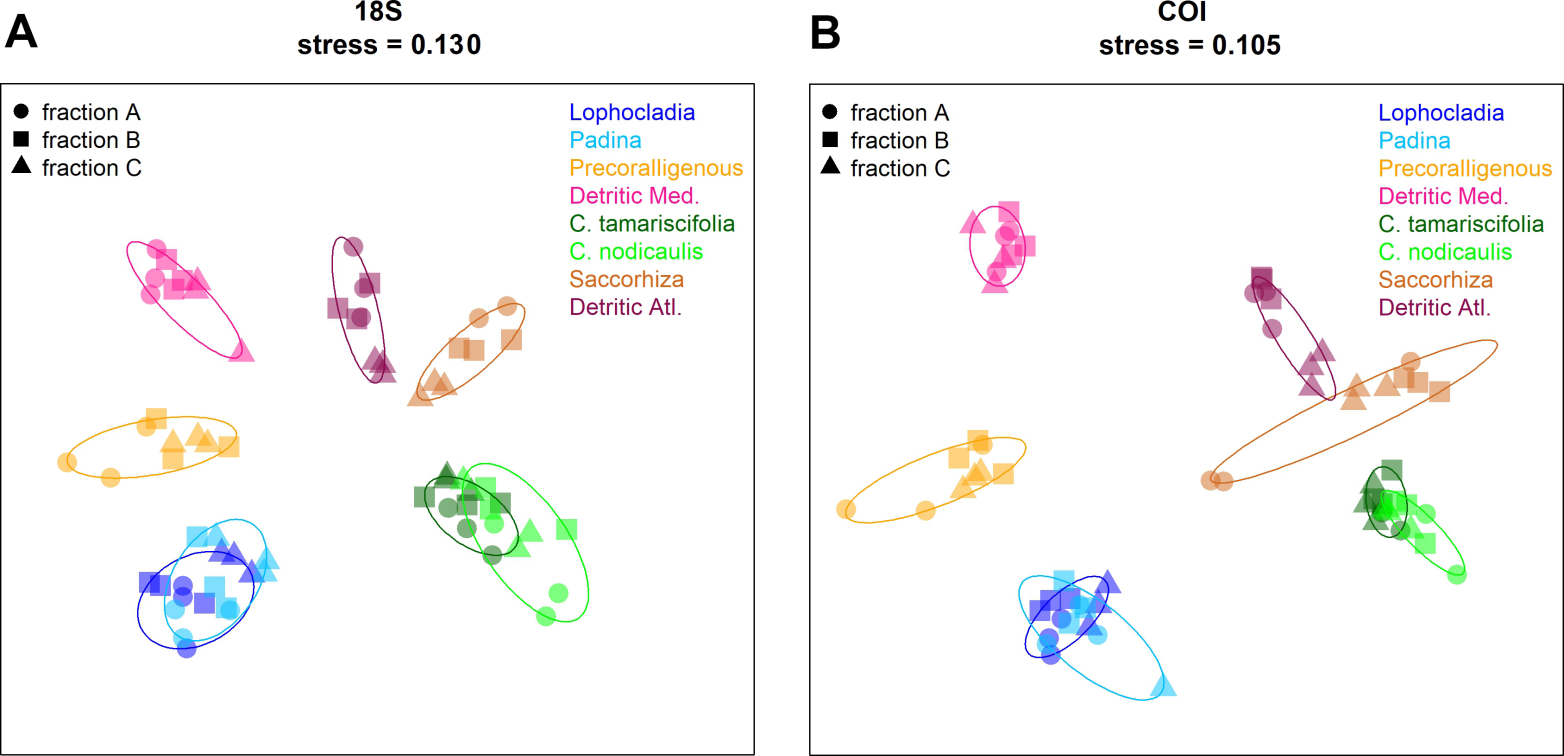
Non-metric multidimensionalscaling (nmMDS) representation of the samples using the Bray–Curtis coefficient. (A) 18S, (B) COI. Samples are symbol-coded for fraction and colour-coded for community. Stress of the final configurations is indicated.

Non-metric multidimensional scaling (nmMDS) plots showing the ordination of the studied communities are shown in Fig. 9 for COI and 18S. Similar ordination patterns were recovered from both markers, and the two Bray–Curtis matrices (18S and COI) were highly correlated (Mantel test, *r* = 0.911, *p* < 0.001). Samples from the three fractions of each community grouped together, with overlap of the inertia ellipses in many cases. Samples from both photophilous atlantic communities clustered together, and the same applies to both photophilous mediterranean communities. On the other hand, mediterranean and atlantic samples appeared clearly separated along the first axis, and a gradient from shallower (well-lit photophilic communities) to deeper, sciaphilous and detritic communities was apparent along the second dimension.

## Discussion

The application of metabarcoding techniques to characterize marine hard bottom communities has been hindered by a lack of standardized methods for sample collection and treatment, the need of improved universal primers capable of amplifying the wide array of taxonomic groups present in these communities, and of bioinformatics procedures able to cope with the high degree of genetic diversity obtained. We think that the procedures presented here, which include extraction of DNA from separate size fractions, a novel set of highly degenerate primers for COI, and improved bioinformatics pipelines for data treatment, including new reference databases for eukaryotes, will help face the challenges related to metabarcoding of structurally complex hard-bottom communities. In this work, we tested this approach on the eukaryotic diversity present in eight ecologically diverse littoral hard-bottom and detritic communities. A similar procedure has already proven useful for detecting effects of three invasive algae on the small-sized organisms of littoral communities in a different set of samples (Wangensteen et al., 2018). We believe that our method can be applied, with the necessary adjustments, for biodiversity assessment in a wide array of marine, terrestrial or freshwater eukaryotic communities.

### Choice of a proper metabarcoding marker, COI vs 18S

The amplification of COI resulted in more MOTUs than 18S (by a factor of 4) and increased taxonomic resolution at the species level (629 vs 376 species-level assignments), at the cost of a higher proportion of MOTUs unassigned at phylum or lower levels (overall 36.5% and 14.7%, respectively), a result consistent with previous findings (e.g., zooplankton, Clarke et al., 2017). Moreover, the assignment at the species level was more reliable using COI than 18S. An assignment with an identity percent higher than 97% using COI leads in general to correct species identification; whereas, in many cases, the assignment of 18S by the ecotag algorithm (even at 100% identity) yielded taxa not present in the studied areas (i.e., common taxa whose geographic distribution is well-known and does not include the northeast Atlantic). This happens because related species included in the reference database share exactly the same sequence for the 18S fragment used, whereas cases of synonymous sequences for different species are extremely rare using COI. Although errors in taxonomic annotation in the databases can also affect species identification, such errors would be present for both markers.

The use of COI as a metabarcoding marker has been criticized in the past, arguing that high rates of sequence variability impair the design of truly universal primers and hamper the bioinformatics analyses (Deagle et al., 2014), but attempts have been made recently to incorporate COI data in metabarcoding studies (e.g., Leray & Knowlton, 2015; Berry et al., 2015; Aylagas et al., 2016; Elbrecht & Leese, 2017). COI presents two major advantages compared to other possible markers: first, the steadily growing international effort to develop a public DNA barcoding database with curated taxonomy, which vastly facilitates taxonomic assignment. The BOLD database (Hebert, Ratnasingham & DeWaard, 2003; Ratnasingham & Hebert, 2007), based mainly in COI barcoding, currently includes over 4 million sequences belonging to more than 500,000 different species, curated and identified by expert taxonomists. It is unlikely that any comparable effort might be undertaken for any other marker in the near future. Second, the high mutation rate of COI practically ensures unequivocal identification at the species level, a resolution that is usually not attainable with the highly conserved sequence of 18S (Tang et al., 2012). Species-level resolution is crucial for calculating ecological indices or detecting non-native species (Comtet et al., 2015; Aylagas et al., 2016).

Overall, we favour the use of COI amplicons for characterizing complex marine communities. The use of 18S is likely adequate when the information at the species level is not crucial. For example, when assessing overall impacts related to human activities such as fisheries, aquaculture or mining facilities (e.g., Keeley, Wood & Pochon, 2018; Laroche et al., 2018), the impact may be expected to affect abundances and composition at higher taxonomical levels. Studying these impacts using 18S may benefit from the less computationally demanding and faster bioinformatics processing of 18S data than that of COI data.

Our universal Leray-XT primer set for COI was able to successfully amplify a wide range of eukaryotic organisms in our samples, belonging to 17 phyla of Metazoa and all major marine lineages of eukaryotes. The undetectability of some minor groups in this study is possibly more related to the incompleteness of reference databases, rather than to primer bias. This modified primer set also showed enhanced coverage *in silico* than the original one. Therefore, although we must acknowledge that a direct comparison of both primer sets, rather than a purely *in silico* assessment, would be necessary, we believe that the new primer set will be useful for COI metabarcoding analyses of marine samples or other environmental or community DNA projects, especially when a wide taxonomic range of eukaryotes is expected and species-level resolution is necessary. We note, however, that these primers have limited ability for detecting some groups (e.g., Viridiplantae and Ciliophora). Thus, specific primers or a different marker should be used if these taxonomic groups constitute the main study target.

It is remarkable that ordination analyses of our data yielded robust and comparable results, disregarding the marker chosen. The two distance matrices were highly correlated, indicating that the same general ecological information is retrieved with both markers. This implies that robust and objective methods for impact studies or comparisons among communities may be designed and implemented with different markers. By contrast, when an accurate taxonomic inventory is the goal, the choice of marker is important, and COI performs better in this respect.

### Taxonomic assignment. Current gaps in databases

We generated reference databases from EMBL for 18S and EMBL plus BOLD for COI with *in silico* ecoPCR (Ficetola et al., 2010) with our metabarcoding primer sets. The rates of unassigned sequences in our results suggest that important gaps still exist for both markers in the genetic repositories, which would prevent the detailed identification of many marine organisms, in agreement with the concerns expressed by other authors (Leray & Knowlton, 2016). Database gaps affect the metazoan groups differentially. For example, despite being abundant and diverse in benthic ecosystems, most bryozoans and cnidarians could be rarely assigned using COI below the class or order level, whereas species of echinoderms, decapods or vertebrates were usually successfully identified at lower levels. A trend can also be seen of smaller sized groups, such as Nematoda, Rotifera or benthic Copepoda, being left out of the databases, whereas bigger sized or commercially important animals such as fish or decapods are well represented. If a fine taxonomic identification of the obtained sequences is desired for a given metabarcoding project, it is advisable not to rely exclusively in the public repositories and obtain custom databases, including the generation of sequences for known local species absent from the repositories.

For many ecological applications, however, it suffices that a particular MOTU is defined, its patterns of distribution and abundance assessed, and changes over time monitored, even if a scientific name for that MOTU is yet unavailable (Cordier et al., 2017). Moreover, the sequences of all MOTUs (identified or not) detected by metabarcoding will remain in public repositories, so that unidentified MOTUs might well be assigned a name in the future, as databases improve. The same can hardly be said of morphological studies, where many taxa cannot be identified to species level either, and are left inventoried under general names (e.g., “Nematoda spp or spA, B, C…”) without descriptions. Therefore, unlike metabarcoding datasets, currently used morphological inventories contain a great deal of untraceable information that can never be used by other researchers at any other place or time.

There is no doubt that taxonomic assignment of COI metabarcoding data will be more accurate and detailed in the future, as reference databases are populated by international barcoding initiatives, such as the Census of Marine Life (http://www.coml.org) or the Marine Barcode of Life (MarBOL, http://www.marinebarcoding.org). We strongly advocate for the continued public support and funding of such collaborative DNA-barcoding projects as a necessary tool towards the implementation of reliable and objective metabarcoding techniques for environmental assessment.

### Sample pre-treatment, the benefits of size fractionation

Although we didn’t test directly unsieved samples of the same communities, the partitioned metabarcoding of size fractions filtered allowed characterization of structurally complex communities at different levels. This characterization would be difficult using whole samples, due to the high number of DNA copies from organisms of bigger biomass outnumbering the smaller ones and hampering their detection (Cowart et al., 2015; Elbrecht, Peinert & Leese, 2017). We have shown that the smallest fractions were the most diverse and were enriched in meiofaunal groups, which can be detected because most of the biomass from big-sized organisms was retained within fractions A and B (Fig. 8). Without size-sorting, these small organisms would probably become rare in the DNA pool, and thus subject to random sequencing issues (Leray & Knowlton, 2017). Therefore, a much higher sequencing depth would be necessary to recover sequences for these groups at frequencies above the filtering thresholds established. Even if there was an important qualitative overlap (Fig. 3), many MOTUs appeared in appreciable abundance only in fraction C.

An additional advantage of this procedure was the removal of a significant fraction of microorganisms (prokaryotes and the smallest microeukaryotes), together with most of the extra-organismal DNA in the form of small remains, cell debris, or extracellular DNA (Creer et al., 2016), which were not retained in the last sieve (63 µm). Microeukaryotes are known to be genetically diverse and under-represented in genetic databases, which adds complexity during clustering and taxonomic assignment steps of bioinformatics analyses. They are better removed from the samples by sieving whenever they are not the main study target. Moreover, many MOTUs with high read abundances could be assigned to the species level using COI, while unassigned MOTUs were typically the least abundant, suggesting again the reference database bias towards big and abundant species. Therefore, in studies mainly aimed at characterizing macro- and meio-benthic components, some physical filtering step is advisable during sample pre-treatment. Size-fractionation has been used to separate relevant compartments in metabarcoding studies of planktonic organisms (e.g., Logares et al., 2014; Massana et al., 2015; Liu et al., 2017) and in some studies of sedimentary bottoms (e.g., Chariton et al., 2010; Cowart et al., 2015; Aylagas et al., 2016). However, this issue had not been addressed for natural hard-bottom benthic communities, where size-differences encompass many orders of magnitude. The closest reference is the study of artificial settlement surfaces (ARMS, Leray & Knowlton, 2015; Ransome et al., 2017) where organisms were separated into sessile biota (processed in bulk) and three size-classes of motile organisms, being the smaller fractions the most diverse. Even if it increases the workload of sample processing, size-fractionation can be recommended for adequate recovery of biodiversity in littoral benthic communities.

### Estimates of *α*-diversity: a comparison with morphological studies

Using 18S, 2,315 different MOTUs were detected in the four mediterranean communities combined, and 2,157 MOTUs in their atlantic counterparts. The respective values for COI MOTU richness were 7,179 for Cabrera and 6,830 for Cíes. Although the values obtained for eukaryotic richness are dependent on the choice of clustering algorithm, they can be considered very high, as the total number of morphological species described for the whole Mediterranean Sea is ca. 17,000 (Coll et al., 2010). Even if we used a conservative pipeline and applied stringent minimal abundance filters to remove low-abundance MOTUs from our final dataset, rarefaction to just 19,000 reads per sample yielded values for MOTU richness of roughly 200–700 MOTUs per sample and fraction for 18S and twice these values for COI. However, an adequate benchmarking of these values against richness detected with traditional (morphology-based) techniques for this kind of communities is still lacking. For sediment macroinvertebrates, Aylagas et al. (2016) showed that the Leray fragment generated over 50% of matches (depending on the protocol and lab conditions tested) between morphologically and molecularly inferred taxonomic composition. We cannot perform such a direct comparison with our samples as morphological information is not available. However, we can draw upon published studies of the same areas and types of community analyzed (see below) to gain an idea of the relative performance of metabarcoding for characterizing biodiversity in hard substratum communities. A more precise benchmarking, analyzing the same samples with both methods, remains to be performed in future studies.

Traditional methods for characterization of these communities rely on randomly allocating sampling units (usually quadrats of 20 × 20 or 25 × 25 cm) and either collecting the biota through scraping, performing *in situ* visual censuses, or analyzing photographs. A comparison of the three methods was made precisely on the Cabrera Archipelago (Sant et al., 2017), highlighting relative differences in outcomes and cost/benefits among methods. However, even the best performing method (scraping) identified a total of 262 species, much lower than we obtained in Cabrera from just the coarser fraction (A): 1,846 MOTUs with 18S (168 identified at species level) and 5,423 with COI (765 identified at species level).

Other studies have analyzed species richness of the macrofauna and macroflora in both National Parks or geographically close areas in communities identical or similar to the ones studied here. In addition, a monograph is available on the taxonomy of benthic groups in Cabrera Archipelago (Alcover, Ballesteros & Fornós, 1993). In File S2 we have collated the information from these works and compared richness values with the ones obtained in our study of the corresponding communities. Metabarcoding largely outperforms morphological inventories, detecting on average ca. 162 and ca. 5 times more MOTUs (COI and 18S, respectively) than reported in exhaustive morphological studies. Only in a few cases (notably Chlorophyta with both markers) did we detect a lower number of MOTUs than morphospecies reported. We must keep in mind that published results are often compilations of several works, while we have results for only a handful of samples taken at a single time point. The dominant genera and species mentioned in quantitative studies are in agreement with the results obtained with metabarcoding, particularly with COI. Our results show that genetic estimates for diversity (especially those obtained from COI metabarcoding) largely exceed the results from morphological assessments, in agreement with other metabarcoding studies which reported higher genetic than morphological diversity estimates in comparable samples (Cowart et al., 2015). This result suggests the existence of a large number of yet undescribed marine taxonomic lineages. Overall, then, metabarcoding seems well suited for biodiversity detection in hard bottoms, with the added advantage that it can target not just macro-organisms as most previous morphological studies did, but also meio- and micro-organisms.

## Conclusions

In this work we showed how complex communities on marine hard-bottoms featuring organisms of a wide range of sizes can be tractable with an adapted community-DNA metabarcoding approach. Size fractionation is highly advisable to adequately capture information for a range of body sizes spanning several orders of magnitude. We assayed a novel primer set for the amplification of the “Leray fragment” of COI, introducing more degenerate positions for increased universality, as shown in *in silico* tests. Results show that COI recovers four times more diversity (in MOTU richness) than sequences of the ribosomal 18S molecule (v7 region). Reference databases were generated from publicly available sequences, showing that significant gaps still prevent complete taxonomic assignment of the sequences. Notwithstanding, assigned (at the phylum or lower level) MOTUs represented >90% of reads for both markers. Our results show that marine metabarcoding, currently applied mostly to plankton or sediments, can be adapted to characterize the bewildering diversity of marine benthic communities dominated by macroscopic seaweeds and colonial or modular sessile metazoans. This expands the range of applications of this technique to ecosystems of enormous ecological and economic importance. At the same time, we have generated the first metabarcoding inventories for natural hard substrate communities, using Marine Protected Areas as our sampling settings, thus providing baseline information for future conservation-oriented research.

##  Supplemental Information

10.7717/peerj.4705/supp-1 File S1 *In silico* evaluation of the new Leray-XT primer set for COI *In silico* amplification results are compared with results obtained with the original Leray primer set for the main metazoan phyla (above, using PrimerMiner) and for the main eukaryotic groups (below, using ecopcr). Primer logos are also shown for the Leray-XT primers.

10.7717/peerj.4705/supp-2 File S2 MOTU richness and morphospecies richness Comparison of MOTU richness values obtained in the present work with morphospecies diversity found with morphological methods in previous studies on the same or similar and geographically close communities. References are listed.

10.7717/peerj.4705/supp-3 Figure S1 Map of the Iberian Peninsula with indication of the two study áreas General map from Google Earth, Image Landsat/Copernicus ©2009 GeoBasis DE/BKG. (A) Atlantic Islands National Park (Google Earth, Image ©2018 Terrametrics). (B) Cabrera Archipelago National Park (Google Earth, Image ©2018 Terrametrics). Yellow dots mark the sampling points.

10.7717/peerj.4705/supp-4 Figure S2 Images of the benthic communities sampled in this study (A-D) Atlantic Islands National Park. (A) photophilous community with *Cystoseira tamariscifolia*, (B) photophilous community with *Cystoseira nodicaulis*, (C) sciaphilous community with *Saccorhiza polyschides*, (D) Atlantic detritic bottoms. (E–F) Cabrera Archipelago National Park, (E) photophilous community dominated by *Lophocladia lallemandii*, (F) photophilous community dominated by *Padina pavonica*, (G) sciaphilous precoralligenous outcrops, and (H) Mediterranean detritic bottoms. In (A) and (D) the quadrat and the corer used for sampling are shown. All pictures by the authors.

10.7717/peerj.4705/supp-5 Figure S3 Values of abundance (in mean n. of reads ±SE) of the MOTUs unassigned and assigned at different taxonomic ranks for both genes

10.7717/peerj.4705/supp-6 Figure S4 Patterns of relative readabundance per community and fraction size. Results obtained using COI (A) or 18S (B) in eight different marine littoral communities from the Atlantic (left) and Mediterranean (right) National Parks studied. Fraction A, coarse; B, intermediate; C, fine. The replicates collected at each community are shown separately.

10.7717/peerj.4705/supp-7 Figure S5 Patterns of relative MOTUrichness per community and fraction size Results obtained using COI (A) or 18S (B) in eight different marine littoral communities from the Atlantic (left) and Mediterranean (right) National Parks studied. Fraction A, coarse; B, intermediate; C, fine. The replicates collected at each community are shown separately.

10.7717/peerj.4705/supp-8 Figure S6 Analysis of replicability for the two genes in a photophilous Atlantic community (*Cystoseira tamariscifolia*) Pie charts represent the relative number of reads obtained for the different groups. Ecological replicates are the three samples collected, one of which was extracted three times separately (extraction replicates) and one of the extractions was PCR-amplified three times (PCR replicates).

10.7717/peerj.4705/supp-9 Table S1 Summary of reference sequences included in thedatabases used for taxonomic assignment of COI and 18S using ecotag

10.7717/peerj.4705/supp-10 Table S2 Pipelines used for theanalyses of the two genes

10.7717/peerj.4705/supp-11 Table S3 MOTU table from 18S Final dataset for 18S, including sequences of all MOTUs, their taxonomic assignment and their abundances in each sample. The most abundant sequence is also presented.

10.7717/peerj.4705/supp-12 Table S4 MOTU table from COI Final dataset for COI, including sequences of all MOTUs, their taxonomic assignment and their abundances in each sample. The most abundant sequence is also presented.

## Additional Information and Declarations

### DNA Deposition

The following information was supplied regarding the deposition of DNA sequences: The original sequences, after pairing and quality checks, are available from the Mendeley data repository: Wangensteen, Owen S.; Palacín, Creu; Guardiola, Magdalena; Turon, Xavier (2018), “Data from: Wangensteen, O.S., Palacín, C., Guardiola, M. & Turon, X. DNA metabarcoding of littoral hard-bottom communities: high diversity and database gaps revealed by two molecular markers”, Mendeley Data, v2.
10.17632/nm2c97fjng.2.

### Data Availability

The following information was supplied regarding data availability: Databases of reference sequences to be used for ecotag taxonomic assignment of COI and 18S are deposited in Github (http://github.com/metabarpark/Reference-databases). R scripts used as part of the analysis pipeline are also deposited in Github (http://github.com/metabarpark/R_scripts_metabarpark).
