# Supplementary material for: DNA metabarcoding of littoral hard-bottom communities: high diversity and database gaps revealed by two molecular markers
Source: PeerJ. 2018 May 4;6:e4705. doi: 10.7717/peerj.4705 (PMC5937484; doi:10.7717/peerj.4705)
Supplement: File S1 — In silico amplification results are compared with results obtained with the original Leray primer set for the main metazoan phyla (above, using PrimerMiner) and for the main eukaryotic groups (below, using ecopcr). Primer logos are also shown for the Leray-XT primers. [file peerj-06-4705-s001.pdf]

Supplementary file S1. *In silico* evaluation of the new Leray-XT primer set for COI

We tested the taxonomic coverage of the new primer set for COI (i.e., its theoretical ability to amplify different taxa of known sequences) and compared the results with those of the original Leray set (Leray et al. 2013) using two different *in silico* approaches. For metazoan phyla, the R package PrimerMiner v0.16 (Elbrecht & Leese 2017) was used, with sequence datasets downloaded from the BOLD database. For the rest of the Eukaryotic groups, we used the ecopcr algorithm and the ecotaxstat function (Ficetola et al. 2010) with sets of sequences downloaded from Genbank.

For the comparison of the metazoan phyla coverage, COI sequences were downloaded from the BOLD database for each metazoan phylum using the batch_download function in package PrimerMiner. Similar sequences (putatively belonging to the same species) were clustered together using the same function and default values (97% identity clustering) to avoid the bias due to over-represented species in the database. Then the centroid sequences of each cluster that were longer than 600 bp were selected and aligned using MAFFT v7 (Katoh & Standley 2013). The alignments were curated by hand to remove clearly divergent sequences. This curating step was necessary, since the automatic batch_download function in PrimerMiner also retrieved some sequences that did not belong to the COI gene. Once aligned, the coverage of the forward primers mlCOIintF-XT and mlCOIintF was evaluated using the function evaluate_primer and default values for mismatch penalties. The percentages of successfully amplified sequences were calculated using function primer_threshold with a threshold value of 200. Only the forward primers were compared, since the reverse primer jgHCO2198 is the same for the two primer sets. The comparative results are shown in Fig. S1.1.

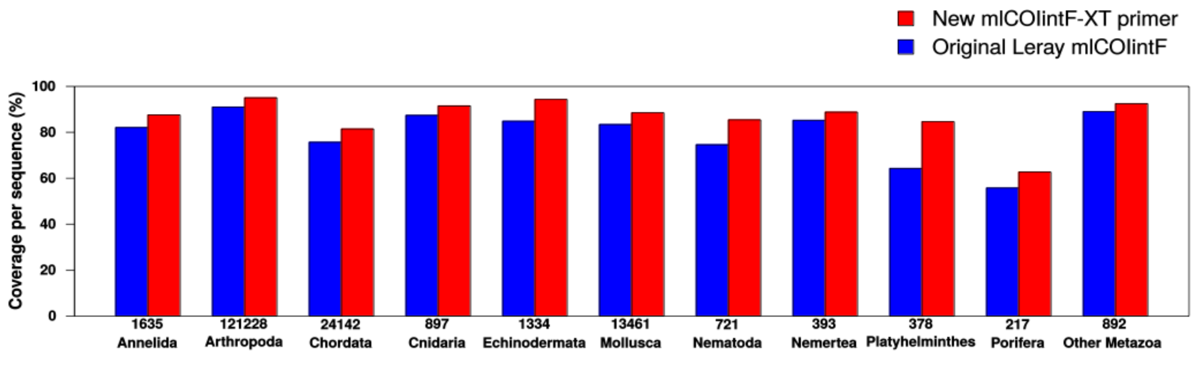

Figure S1.1. *In silico* coverage (percentage of sequences with successful amplification) predicted by PrimerMiner for the new primer mlCOIintF-XT compared to the original Leray mlCOIintF on different metazoan phyla. Numbers in the horizontal axis indicate the total number of sequences evaluated for each phylum.

Even though the results of PrimerMiner evaluations are a conservative estimation of the real ability of the primers to amplify target sequences, our results showed significant improvements for the new mlCOIintF-XT compared to the original Leray primer, with enhancements that ranged from 4.2% (Nemertea) to 31.7% (Platyhelminthes). The average value was 9.8% increase of the number of sequences successfully amplified by the original mlCOIintF. The most enhanced phyla were Platyhelminthes, Nematoda, Porifera and Echinodermata, whereas other phyla which already had high coverage values with the original Leray primer showed smaller enhancements.

The PrimerMiner workflow is not useful for taxa which are not well represented in the BOLD database, since the automatic query to Genbank implemented in batch_download retrieves too many sequences not belonging to the desired fragment. So, for the rest of eukaryotic groups, the ability of each primer set to amplify the different targets was assessed using the *in silico* PCR approach implemented in ecopcr and the ecotaxstat function to evaluate the percentage of species in the group that could be successfully amplified (Ficetola et al. 2010), allowing three mismatches per primer but requiring two perfect matches in the 3' end. In this approach, there is no need to cluster the sequences, since the ecotaxstat function uses the taxonomic information from the queried dataset to calculate the percentage of the present species that can be successfully amplified by the primer, thus avoiding the possible bias due to over-represented species. However, this approach cannot be used directly with partial COI sequences (corresponding to the standard barcoding region), since these barcodes lack the reverse primer binding sequence needed by ecopcr. Thus, for most eukaryotic groups, we used datasets including only complete sequences of COI retrieved from Genbank. However, for those eukaryotic groups where not enough complete COI sequences were available (Dinoflagellata, Rhodophyta and Stramenopiles), we ran ecopcr and ecotaxstat against sets of COI barcode sequences with an artificial jgHCO2198-matching sequence attached to the 3' end. This still allowed us to compare the coverages of the two internal forward primers, since the reverse primer is shared by both sets.

The results for several groups of Eukaryota are shown in Fig. S1.2. The predicted coverage of the new mlCOIintF-XT primer was enhanced for most eukaryotic groups compared to the original Leray primer. The coverage was higher than 96% for all major groups analyzed, except for Fungi, Viridiplantae and Ciliophora. With the exception of Amoebozoa and Rhodophyta (that had already coverage values close to 100% with the original Leray primer set), the new primer mlCOIintF-XT produced significant enhancements in the predicted coverage (5.6% better for Stramenopiles, around 11% better for Dinoflagellata, Fungi and Metazoa, 27% better for Apicomplexa and a 79% increase in Viridiplantae, although the values for Fungi and Viridiplantae still cannot be considered universal. However, none of the seven available complete COI sequences for Ciliophora could be predictably amplified by neither of the tested primer sets.

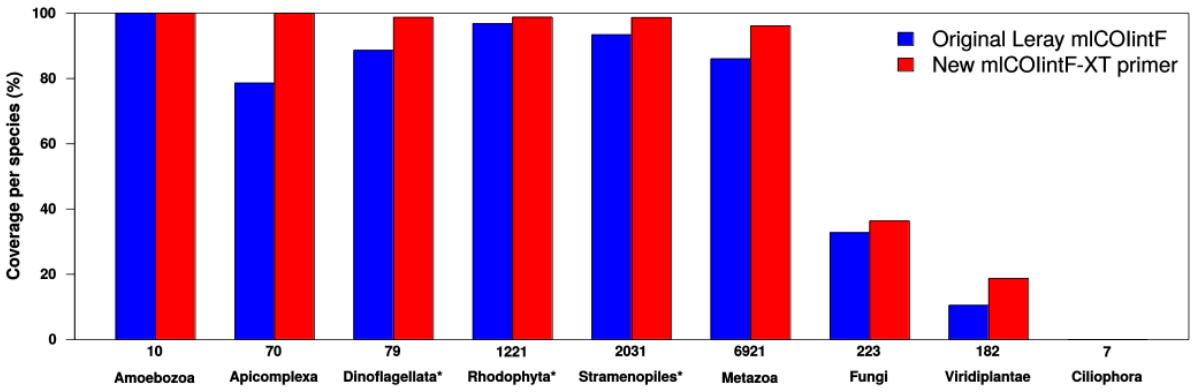

Figure S1.2. *In silico* coverage (percentage of species with successful amplification) predicted by ecopcr for the new primer mlCOIintF-XT compared to the original Leray mlCOIintF on different eukaryotic groups. Numbers in the horizontal axis indicate the total number of complete sequences for COI evaluated for each group, except for groups marked with * in which partial sequences for COI were used, including the forward primer binding site and with artificial reverse primer sequences attached.

Finally, primer logos (Crooks et al. 2004) were obtained to summarize conservation of primer sequences across all eukaryotic groups (Fig. S1.3). These primer logos were drawn from a combined set of 148,908 COI sequences from 38 different phyla of all eukaryotic lineages, that could be amplified *in silico* by ecopcr against release r117 of the EMBL nucleotide database.

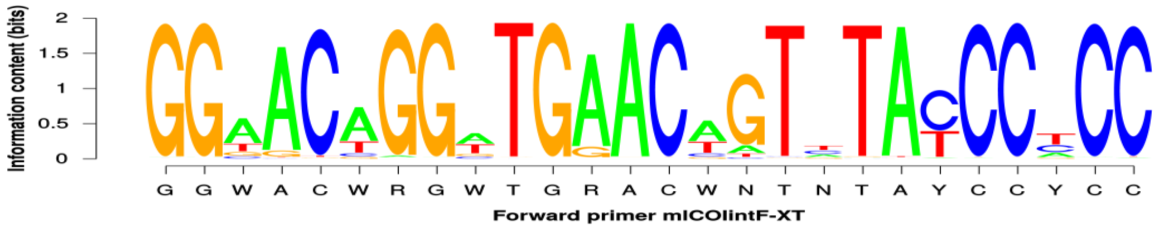

Figure S1.3. Primer logos for the Leray-XT primer set for COI. Generated from 148,908 sequences belonging to 38

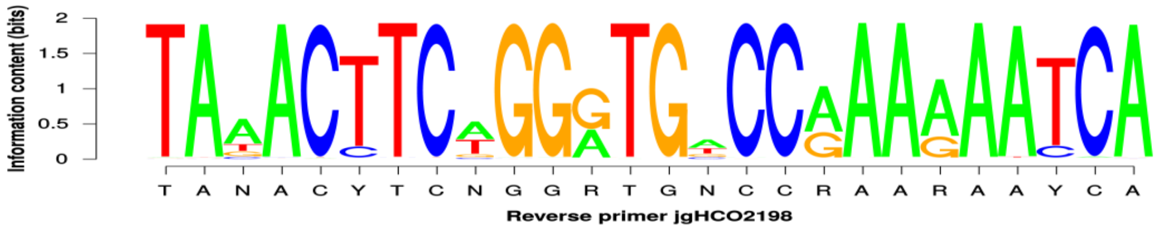

different phyla of all eukaryotic lineages.

References

Crooks GE, Hon G, Chandonia JM, Brenner SE (2004) WebLogo: a sequence logo generator. *Genome Research*, **14**, 1188–1190.

Elbrecht V, Leese F (2017) PrimerMiner: an R package for development and in silico validation of DNA metabarcoding primers. *Methods in Ecology and Evolution,* **8**, 622-626.

Ficetola GF, Coissac E, Zundel S et al. (2010) An in silico approach for the evaluation of DNA barcodes. *BMC Genomics*, **11**, 434.

Katoh K, Standley DM (2013) MAFFT multiple sequence alignment software version 7: improvements in performance and usability. *Molecular Biology and Evolution*, **30**, 772-780.
