# Supplementary material for: DNA metabarcoding of littoral hard-bottom communities: high diversity and database gaps revealed by two molecular markers
Source: PeerJ. 2018 May 4;6:e4705. doi: 10.7717/peerj.4705 (PMC5937484; doi:10.7717/peerj.4705)
Supplement: File S2 — Comparison of MOTU richness values obtained in the present work with morphospecies diversity found with morphological methods in previous studies on the same or similar and geographically close communities. References are listed. [file peerj-06-4705-s002.pdf]

Supplementary file S2. MOTU richness and morphospecies richness. Comparison of MOTU richness values obtained in the present work with morphospecies diversity found with morphological methods in previous studies on the same or similar and geographically close communities. References are listed.

Studied area	Community	Group	Morphospecies richness	MOTU richness 18S	MOTU richness COI	References
Ria de Vigo	Cíes detritic	Rhodophyta<br>Phaeophyceae<br>Chlorophyta	123<br>19<br>18	203<br>31<br>5	411<br>79<br>5	Peña & Bárbara 2008
NW Portugal	Cíes *C. tamariscifolia*	Rhodophyta<br>Phaeophyceae<br>Chlorophyta<br>Polychaeta<br>Crustacea<br>Mollusca<br>Echinodermata	30<br>12<br>4<br>45<br>35<br>9<br>4	132<br>40<br>3<br>176<br>217<br>102<br>16	198<br>99<br>4<br>211<br>627<br>381<br>9	Marques et al. 1982
Balearic Islands	Cabrera precoralligenous	Rhodophyta<br>Phaeophyceae<br>Chlorophyta	85<br>12<br>5	176<br>34<br>3	319<br>51<br>3	Ballesteros 1994
Mediterranean Spain	Cabrera detritic	Rhodophyta<br>Phaeophyceae<br>Chlorophyta	197<br>44<br>31	186<br>27<br>5	347<br>45<br>4	Peña & Bárbara 2008
Cabrera archipelago	Cabrera all communities	Macroalgae<br>Porifera<br>Cnidaria<br>Bryozoa<br>Decapoda<br>Mollusca<br>Ascidiacea<br>Echinodermata	277<br>98<br>85<br>145<br>69<br>169<br>41<br>53	303<br>154<br>177<br>183<br>27<br>105<br>42<br>16	812<br>473<br>862<br>469<br>26<br>239<br>74<br>16	Ballesteros 1993<br>Uriz 1993<br>Gili et al. 1993<br>Zabala 1993<br>Corbera et al. 1993<br>Altaba 1993<br>Turon 1993<br>Munar 1993

References:

Altaba CR (1993) Els mol.luscs marins: catàleg preliminar. In: Alcover JA, Ballesteros E, Fornós JJ, editors. *Història Natural de l'Archipèlag de Cabrera*. Palma de Mallorca, Spain. Editorial Moll-CSIC, 503-530.

Ballesteros E (1993) Algues bentòniques i fanerògames marines. In: Alcover JA, Ballesteros E, Fornós JJ, editors. *Història Natural de l'Archipèlag de Cabrera*. Palma de Mallorca, Spain. Editorial Moll-CSIC, 503-530.

Ballesteros E (1994) The deep-water *Peyssonnelia* beds from the Balearic Islands (Western Mediterranean). *PSZNI Marine Ecology*, **15**, 233-253.

Corbera J, Ballesteros E, Garcia Ll (1993) Els crustacis decàpodes. In: Alcover JA, Ballesteros E, Fornós JJ, editors. *Història Natural de l'Archipèlag de Cabrera*. Palma de Mallorca, Spain. Editorial Moll-CSIC, 579-587.

Gili JM, Garcia-Rubies A, Tur JM (1993) Els cnidaris bentònics. In: Alcover JA,

Ballesteros E, Fornós JJ, editors. *Història Natural de l'Archipèlag de Cabrera*. Palma de Mallorca, Spain. Editorial Moll-CSIC, 549-559.

Marques VM, Reis CS, Calvário J, Marques JC, Melo R, Santos R (1982) Contribuçao para o estudo dos povoamentos bentónicos (substrato rochoso) da costa occidental portuguesa. Zona intertidal. *Oecologia Aquatica*, **6**, 119-145.

Munar J (1993) Els equinoderms. In: Alcover JA, Ballesteros E, Fornós JJ, editors. *Història Natural de l'Archipèlag de Cabrera*. Palma de Mallorca, Spain. Editorial Moll-CSIC, 597-606.

Peña V, Bárbara I (2008) Maërl community in the north-western Iberian Peninsula: a review of floristic studies and long-term changes. *Aquatic Conservation: Marine and Freshwater Ecosystems*, **18**, 339-366.

Turon X (1993) Els ascidis: faunística i distribució. In: Alcover JA, Ballesteros E, Fornós JJ, editors. *Història Natural de l'Archipèlag de Cabrera*. Palma de Mallorca, Spain. Editorial Moll-CSIC, 607-621.

Uriz MJ (1993) Les esponges litorals. In: Alcover JA, Ballesteros E, Fornós JJ, editors. *Història Natural de l'Archipèlag de Cabrera*. Palma de Mallorca, Spain. Editorial Moll-CSIC, 531-547.

Zabala M (1993) Els briozous. In: Alcover JA, Ballesteros E, Fornós JJ, editors. *Història Natural de l'Archipèlag de Cabrera*. Palma de Mallorca, Spain. Editorial Moll-CSIC, 561-577.
