# Supplementary figures and images for: DNA metabarcoding of littoral hard-bottom communities: high diversity and database gaps revealed by two molecular markers
Source: PeerJ. 2018 May 4;6:e4705. doi: 10.7717/peerj.4705 (PMC5937484; doi:10.7717/peerj.4705)

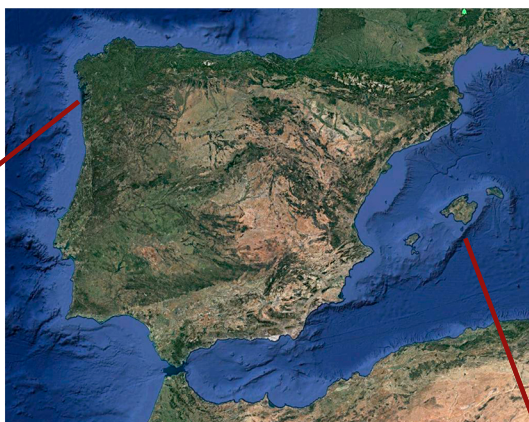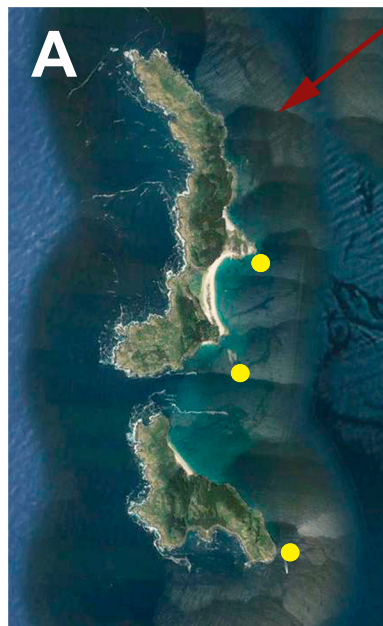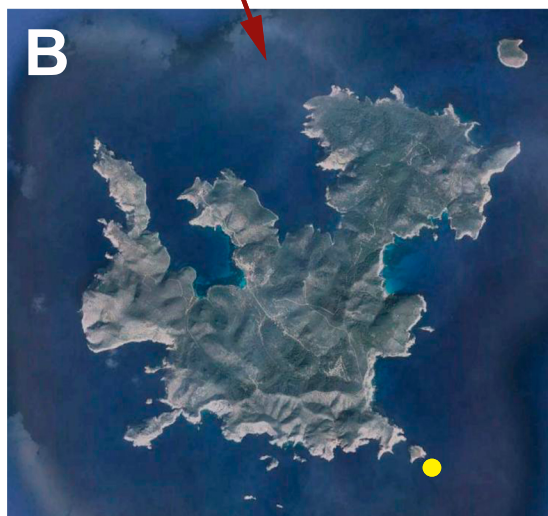

Supplement: Figure S1 — General map from Google Earth, Image Landsat/Copernicus ©2009 GeoBasis DE/BKG. (A) Atlantic Islands National Park (Google Earth, Image ©2018 Terrametrics). (B) Cabrera Archipelago National Park (Google Earth, Image ©2018 Terrametrics). Yellow dots mark the sampling points. [file peerj-06-4705-s003.pdf]

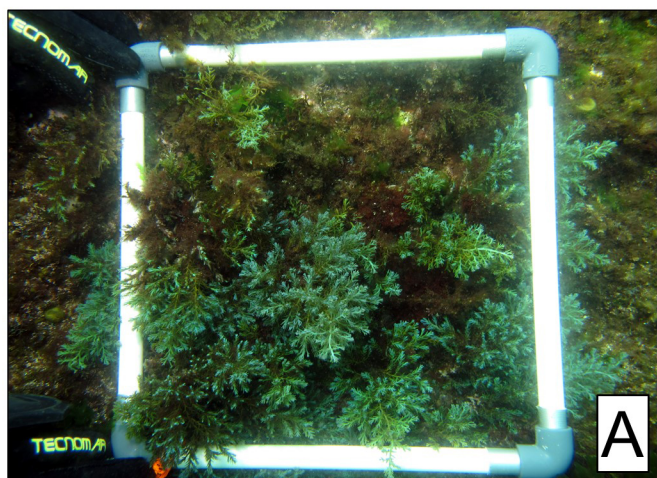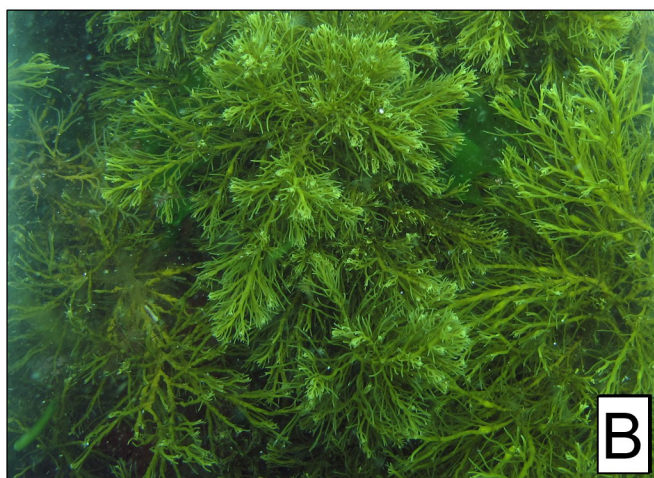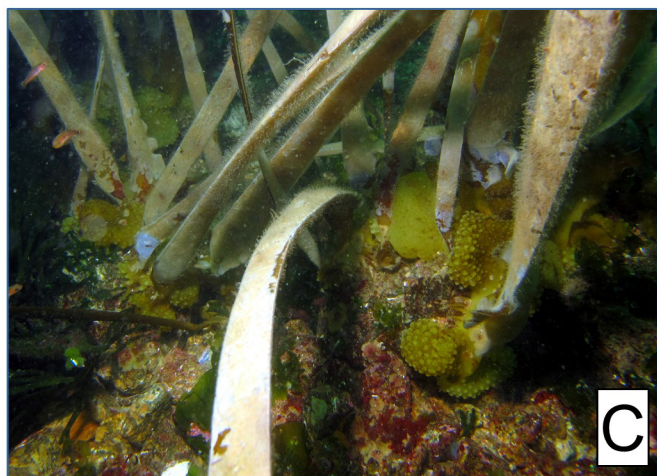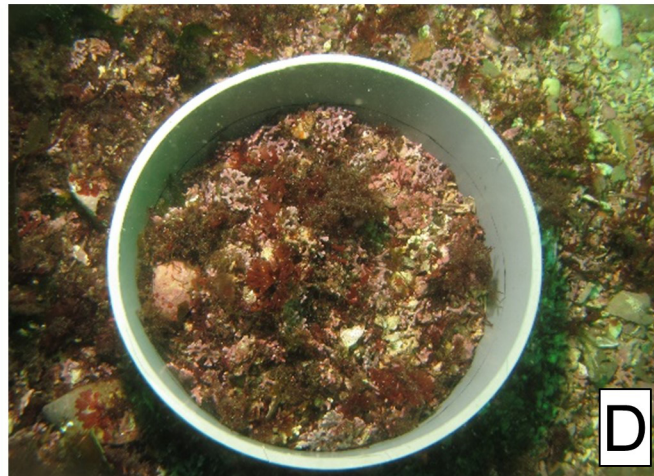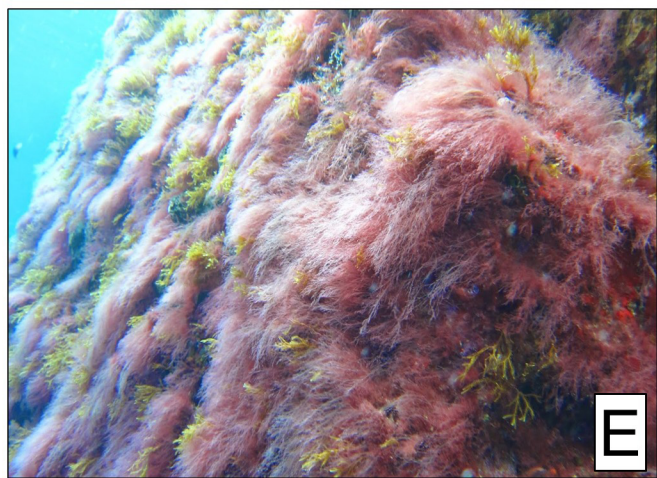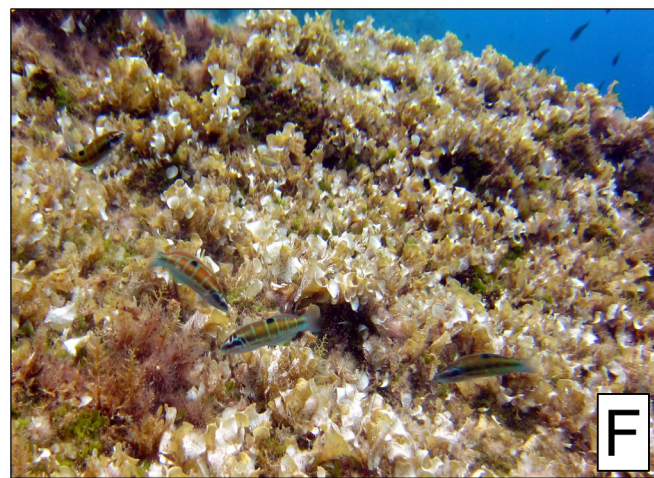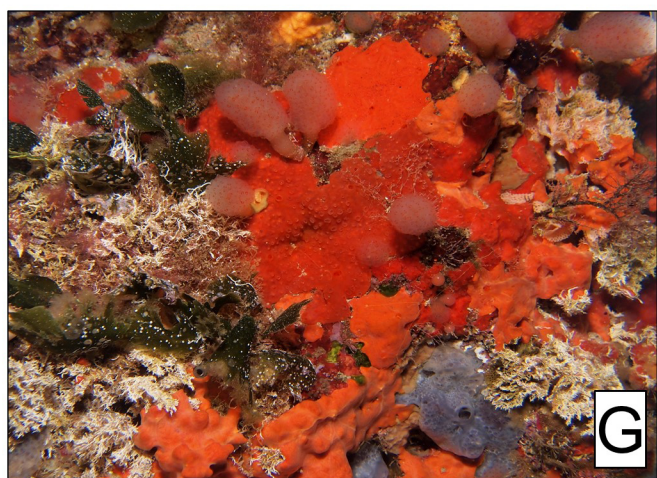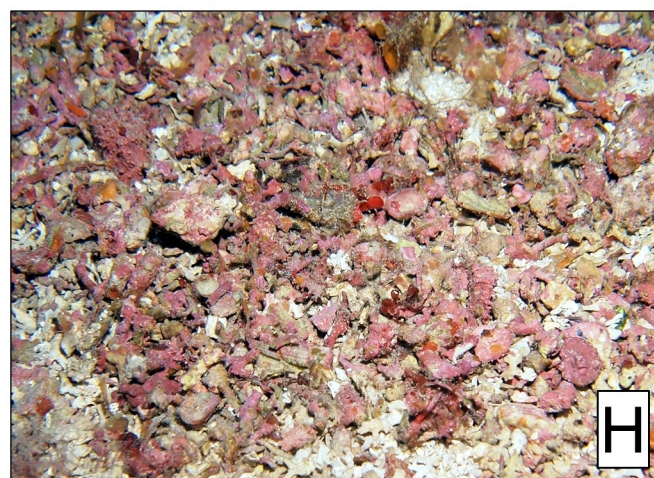

Supplement: Figure S2 — (A-D) Atlantic Islands National Park. (A) photophilous community with Cystoseira tamariscifolia, (B) photophilous community with Cystoseira nodicaulis, (C) sciaphilous community with Saccorhiza polyschides, (D) Atlantic detritic bottoms. (E–F) Cabrera Archipelago National Park, (E) photophilous community dominated by Lophocladia lallemandii, (F) photophilous community dominated by Padina pavonica, (G) sciaphilous precoralligenous outcrops, and (H) Mediterranean detritic bottoms. In (A) and (D) the quadrat and the corer used for sampling are shown. All pictures by the authors. [file peerj-06-4705-s004.pdf]

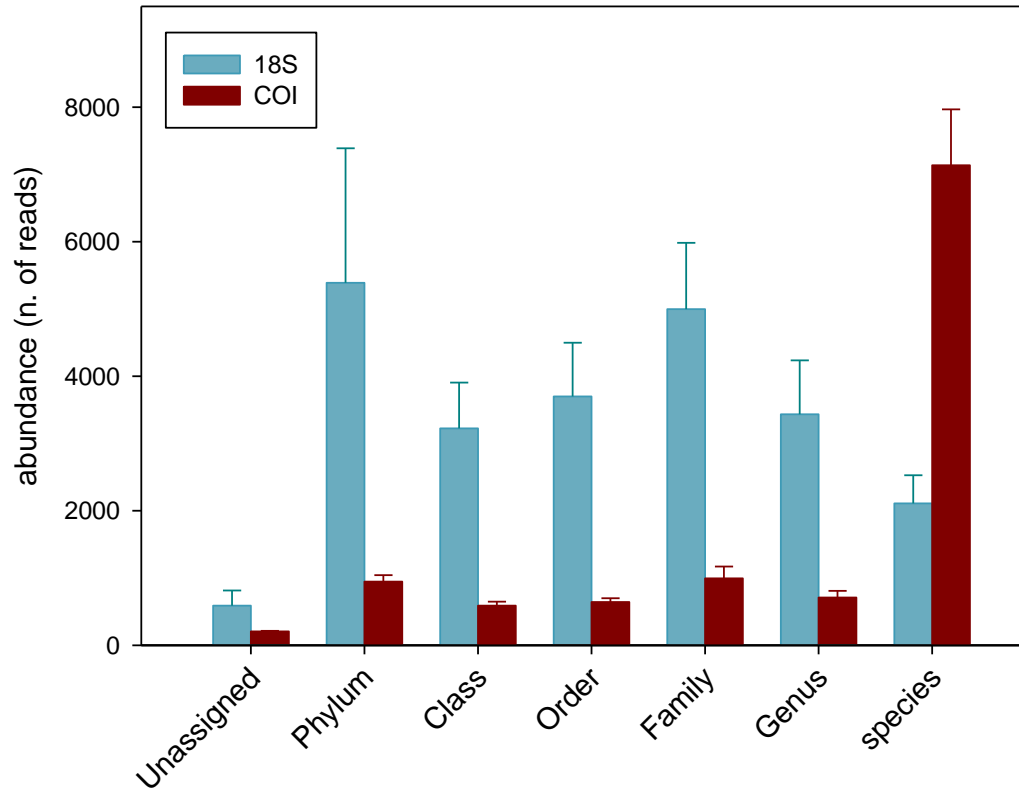

Supplement: Figure S3 [file peerj-06-4705-s005.pdf]

**A**

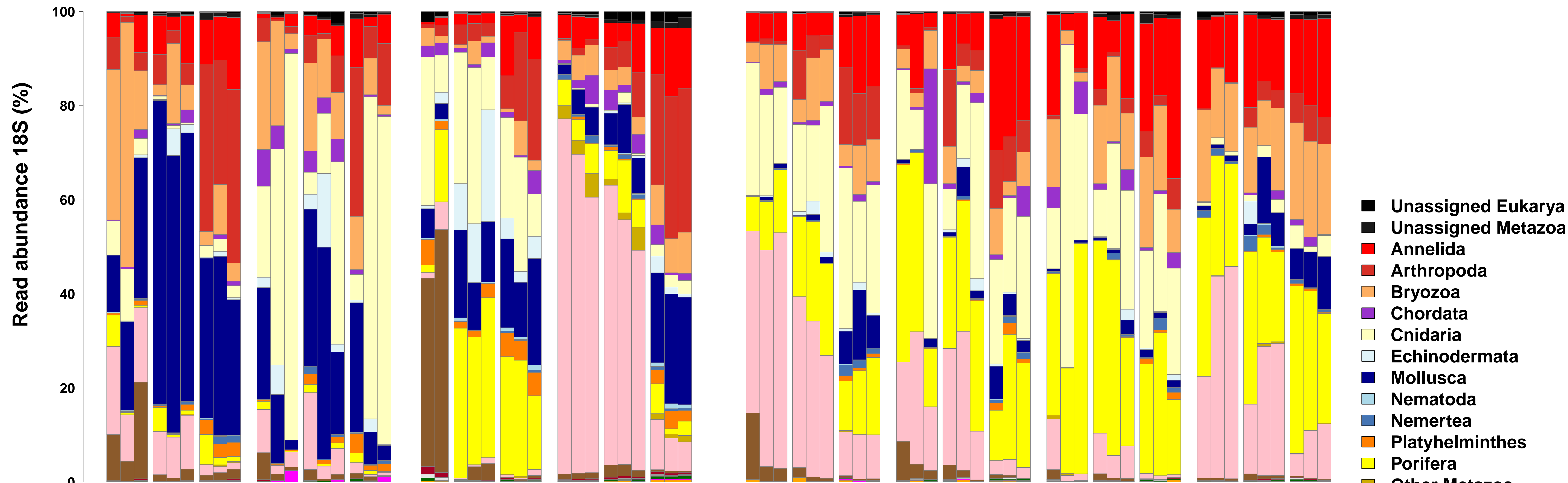

**B**

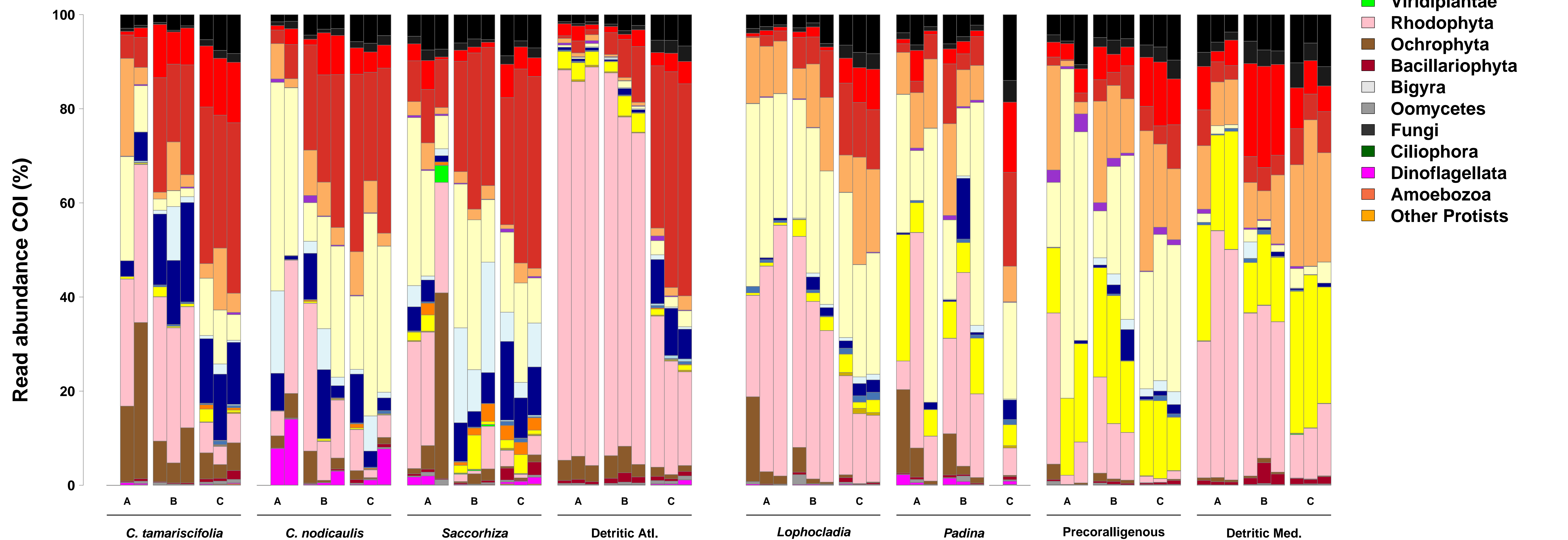

Supplement: Figure S4 — Results obtained using COI (A) or 18S (B) in eight different marine littoral communities from the Atlantic (left) and Mediterranean (right) National Parks studied. Fraction A, coarse; B, intermediate; C, fine. The replicates collected at each community are shown separately. [file peerj-06-4705-s006.pdf]

**A**

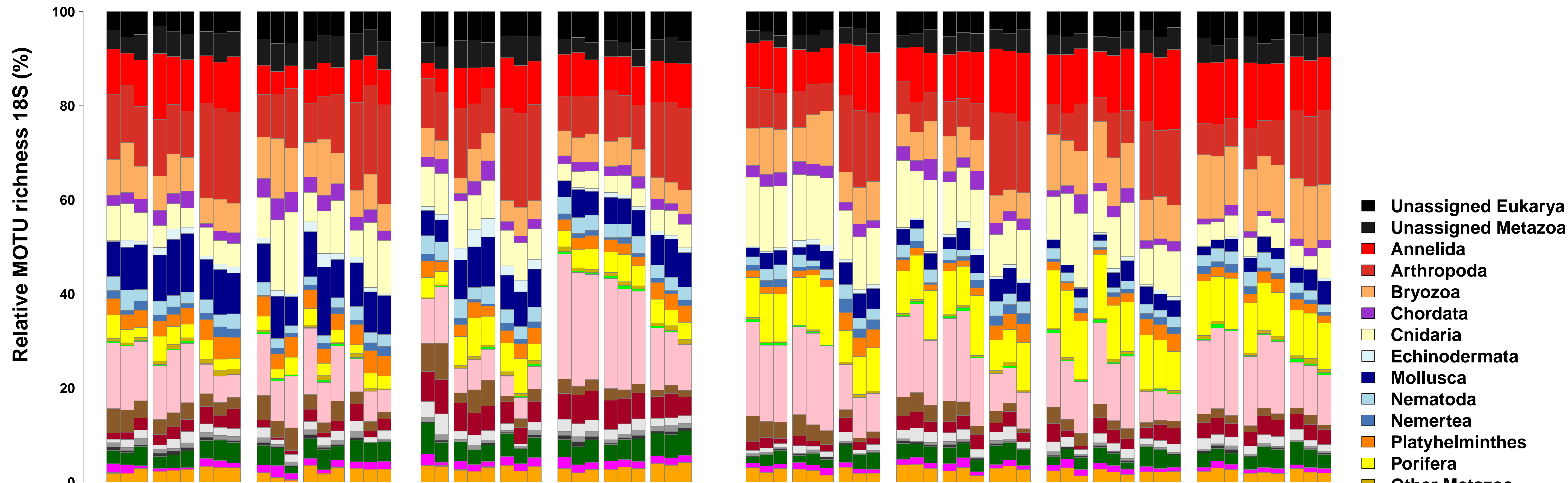

**B**

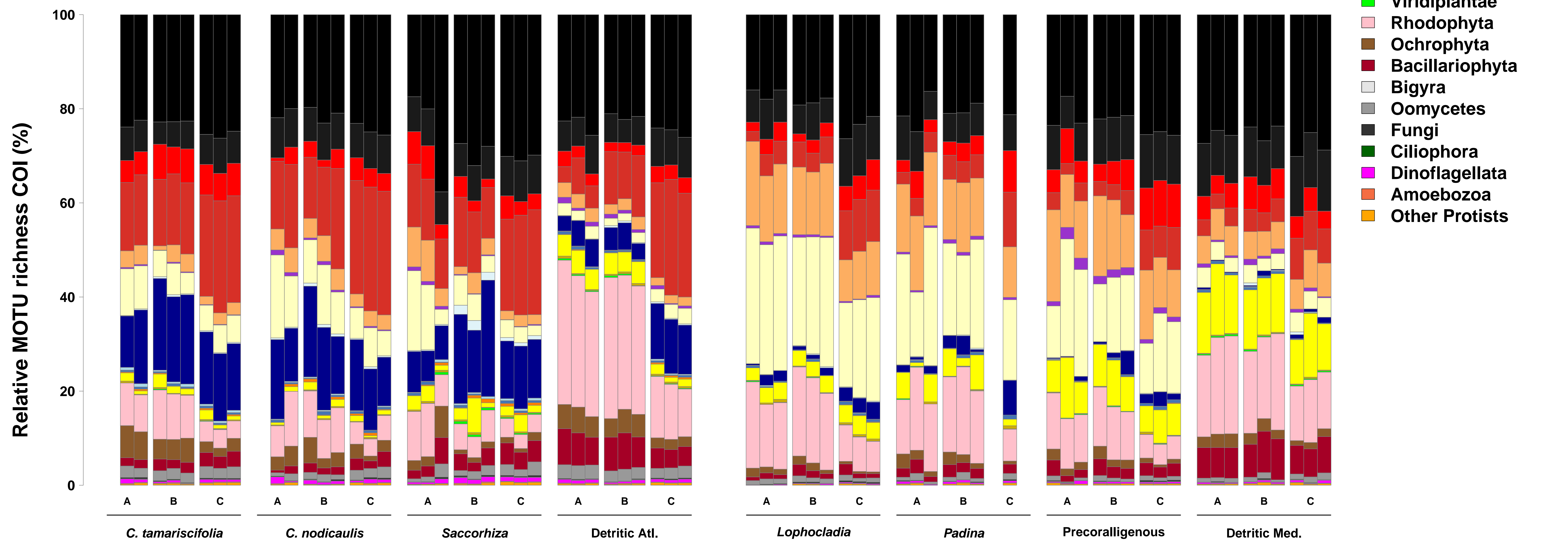

Supplement: Figure S5 — Results obtained using COI (A) or 18S (B) in eight different marine littoral communities from the Atlantic (left) and Mediterranean (right) National Parks studied. Fraction A, coarse; B, intermediate; C, fine. The replicates collected at each community are shown separately. [file peerj-06-4705-s007.pdf]

**18S**

**ecological replicates**

**COI**

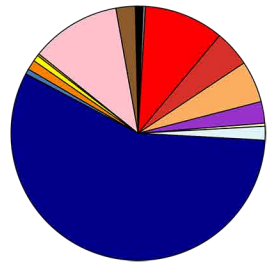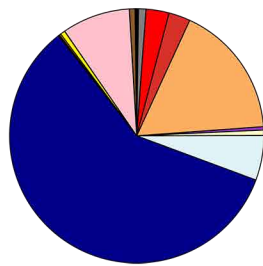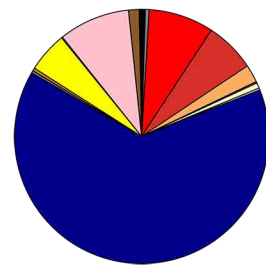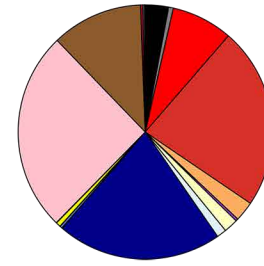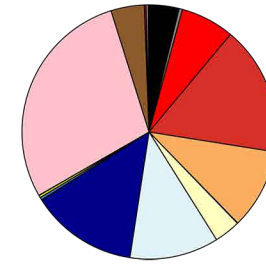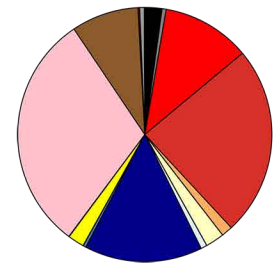

**extraction replicates**

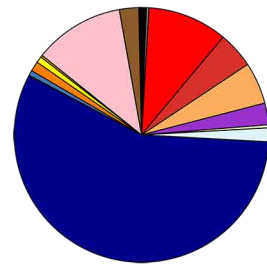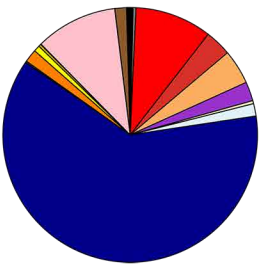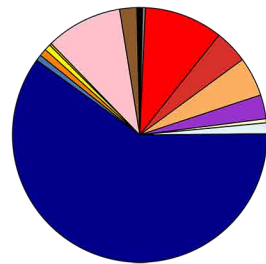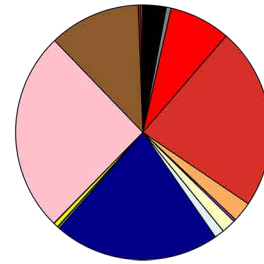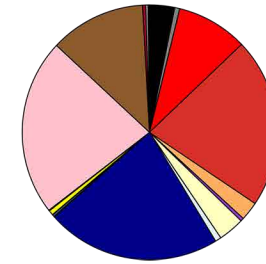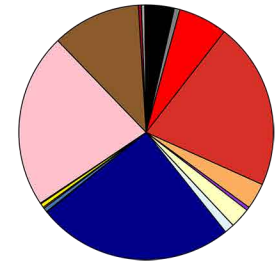

**PCR replicates**

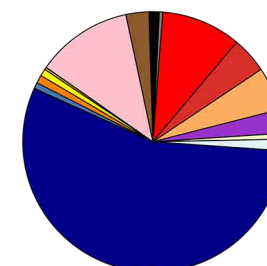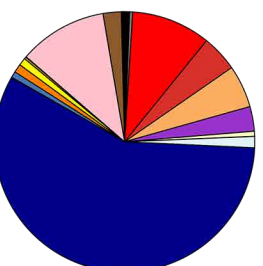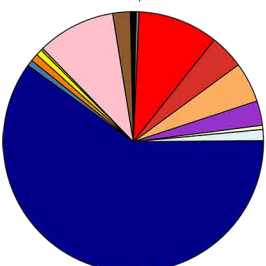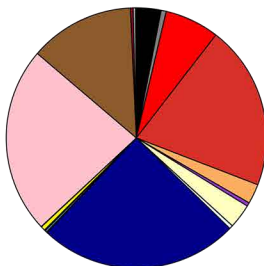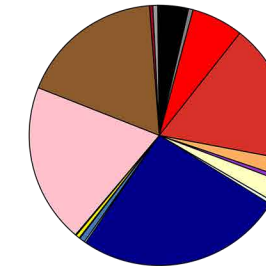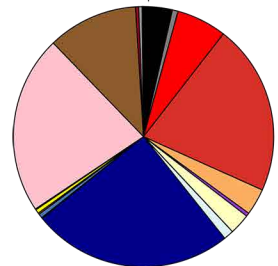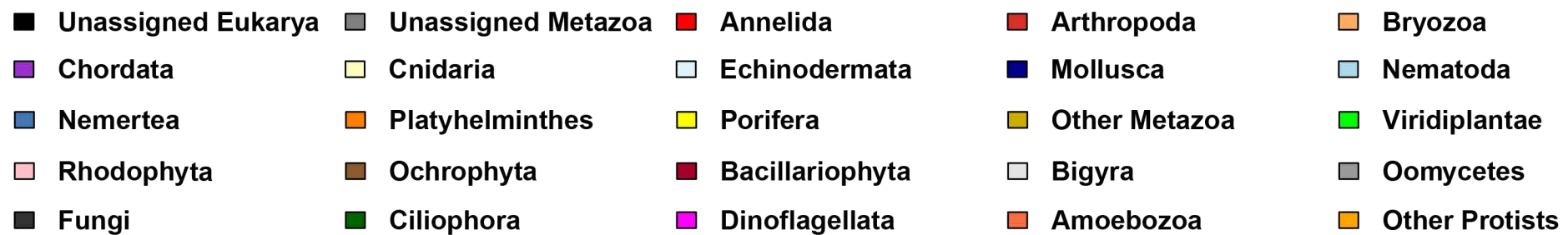

Supplement: Figure S6 — Pie charts represent the relative number of reads obtained for the different groups. Ecological replicates are the three samples collected, one of which was extracted three times separately (extraction replicates) and one of the extractions was PCR-amplified three times (PCR replicates). [file peerj-06-4705-s008.pdf]
