# Supplementary material for: DNA metabarcoding of littoral hard-bottom communities: high diversity and database gaps revealed by two molecular markers
Source: PeerJ. 2018 May 4;6:e4705. doi: 10.7717/peerj.4705 (PMC5937484; doi:10.7717/peerj.4705)
Supplement: Table S1 [file peerj-06-4705-s009.pdf]

Table S1. Summary of reference sequences included in the databases used for taxonomic assignment of COI and 18S using ecotag.

**Taxonomic group**	**Number of reference sequences in db_COI_MBPK database**	**Number of reference sequences in db_18S_r117 database**
Prokaryota	45	2
Amoebozoa	16	462
Apicomplexa	166	628
Apusozoa	0	22
Centroheliozoa	0	36
Ciliophora	99	904
Cryptophyta	5	178
Dinoflagellata	603	423
Excavata Euglenozoa	6	475
Excavata Other	11	149
Fungi Ascomycota	156	1200
Fungi Basidiomycota	25	584
Fungi Blastocladiomycota	2	29
Fungi Chytridiomycota	4	62
Fungi Cryptomycota	0	1
Fungi Entomophthoromycota	0	48
Fungi Entorrhizomycota	0	4
Fungi Glomeromycota	1	122
Fungi Microsporidia	0	60
Fungi Neocallimastigomycota	0	11
Fungi Zygomycota	5	195
Glaucophyta	6	4
Haptophyta	9	94
Metazoa Acanthocephala	95	59
Metazoa Annelida	7269	1018
Metazoa Arthropoda	108843	8275
Metazoa Brachiopoda	44	34
Metazoa Bryozoa	158	137
Metazoa Chaetognatha	44	20
Metazoa Chordata	40037	339
Metazoa Cnidaria	1118	671
Metazoa Ctenophora	1	12
Metazoa Echinodermata	4771	66
Metazoa Entoprocta	3	17
Metazoa Gastrotricha	52	95
Metazoa Gnathostomulida	2	24
Metazoa Hemichordata	4	17
Metazoa Kinorhyncha	0	23
Metazoa Loricifera	0	2
Metazoa Mollusca	15032	858
Metazoa Myzostomida	1	0
Metazoa Nematoda	1507	1547
Metazoa Nematomorpha	0	13
Metazoa Nemertea	160	79
Metazoa Onychophora	363	38
Metazoa Platyhelminthes	1439	1259
Metazoa Porifera	397	244
Metazoa Priapulida	4	5
Metazoa Rhombozoa	14	4
Metazoa Rotifera	761	34
Metazoa Tardigrada	229	109
Metazoa Xenacoelomorpha	60	134
Opisthokonta Choanozoa	0	52
Perkinsozoa	0	8

Rhizaria Cercozoa	2	193
Rhizaria Foraminifera	0	948
Rhizaria Polycystinea	0	84
Rhizaria Other	0	2
Rhodophyta	4723	688
Stramenopiles Bacillariophyta	68	584
Stramenopiles Chrysophyceae	7	72
Stramenopiles Bigyra	2	101
Stramenopiles Eustigmatophyceae	8	25
Stramenopiles Oomycetes	235	70
Stramenopiles Phaeophyceae	170	65
Stramenopiles Raphidophyceae	26	5
Stramenopiles Synurophyceae	2	45
Stramenopiles Xanthophyceae	8	30
Stramenopiles Other	0	22
Viridiplantae Chlorophyta	28	717
Viridiplantae Streptophyta	128	1588
**TOTAL**	**188929**	**26125**
