# Supplementary material for: DNA metabarcoding of littoral hard-bottom communities: high diversity and database gaps revealed by two molecular markers
Source: PeerJ. 2018 May 4;6:e4705. doi: 10.7717/peerj.4705 (PMC5937484; doi:10.7717/peerj.4705)
Supplement: Table S2 [file peerj-06-4705-s010.pdf]

Table S2. Summary of the metabarcoding pipelines for 18S and COI.

METABARPARK 18S PIPELINE

**Process**	**Software**	**Total reads**	**Number of clusters/MOTUs**
QC of Raw sequences	`fasttqc`	2 x 15,121,913	
Paired-end alignment	`illuminapairedend`	15,121,913	
Post-alignment QC	`obiannotate score>40`<br>`obisplit`	14,690,851	
Demultiplexing	`ngsfilter`	8,718,756	
Group the unique seqs	`obiuniq`	8,718,756s	440,862
Delete singletons	`obigrep count>1`	8,403,738	125,844
Sequence length filter 75-180 bp	`obigrep seq_length>75 &`<br>`seq_length<180`	8,372,867	122,303
Clustering at 99%	`CROP l=0.3 u=0.5`<br>`obitab`<br>`RECOUNT.AFTER.CROP.R`	8,372,867	7,145
Annotate with Ecotag	`ecotag db_18s_r117.fasta`<br>`owi_add_taxonomy`	8,372,867	7,145
Blank correction	`Rscript`<br>`Metabarpark_18S.calculations.R`	8,356,242	7,090
Delete unassigned MOTUs with <10 reads		8,351,630	5,897
Delete all MOTUs with < 5 reads		8,349,530	5,067
Delete defective samples (< 10,000 reads)		8,344,298	5,067
Abundance renormalization	`owi_renormalize`	8,266,952	5,067
Minimum relative abundance filter per sample = 0.01%	`Rscript`<br>`Remove_MOTUs_0.01`	7,640,737	2,510

METABARPARK COI PIPELINE

**Process**	**Software**	**Total reads**	**Number of clusters/MOTUs**
QC of Raw sequences	`fasttqc`<br>`fastx_trimmer`	2 x 15,280,243	
Paired-end alignment	`illuminapairedend`	15,280,243	
Post-alignment QC	`obiannotate`<br>`obisplit`	14,338,344	
Demultiplexing	`ngsfilter`	11,806,729	
Sequence length filter 300-320 bp	`obigrep`	11,302,626	
Group the unique seqs	`obiuniq`	11,302,626	3,735,669
Rename the identifiers	`obiannotate`	11,302,626	3,735,669
Chimera removal	`VSEARCH uchime_denovo`	11,142,197	3,609,834
Clustering at 95%	`CROP l=1.5 u=2.5 z=400`<br>`obitab`<br>`owi_recount_crop.R`	11,142,197	63,724
Taxonomic identification	`ecotag db_COI_MBPK`<br>`owi_add_taxonomy`	11,142,197	63,724
Blank correction and removal of Prokaryota	`Rscript`<br>`Metabarpark_COI.blank.R`	10,266,592	52,750
Collapse duplicated species	`owi_collapse`	10,266,592	39,777
Delete unassigned MOTUs with <10 reads	`Rscript`<br>`Metabarpark_COI.calculations.R`	10,204,250	26,054
Delete all MOTUs with < 5 reads		10,188,565	21,453
Delete defective samples (< 10,000 reads)		10,171,101	21,452
Abundance renormalization	`owi_renormalize`	10,093,453	21,452
Minimum relative abundance filter per sample = 0.01%	`Rscript`<br>`Remove_MOTUs_0.01`	9,128,836	9,679
